# Engineering mechanisms of proton-coupled electron transfer to a titanium-substituted polyoxovanadate–alkoxide†

**DOI:** 10.1039/d4sc06468b

**Published:** 2025-01-07

**Authors:** Shannon E. Cooney, S. Genevieve Duggan, M. Rebecca A. Walls, Noah J. Gibson, James M. Mayer, Pere Miro, Ellen M. Matson

**Affiliations:** a Department of Chemistry, University of Rochester Rochester NY 14627 USA matson@chem.rochester.edu; b Department of Chemistry, University of Iowa Iowa City IA 52240 USA; c Department of Chemistry, University of South Dakota Vermillion SD 57069 USA; d Department of Chemistry, Yale University New Haven Connecticut 06520 USA

## Abstract

Metal oxides are promising catalysts for small molecule hydrogen chemistries, mediated by interfacial proton-coupled electron transfer (PCET) processes. Engineering the mechanism of PCET has been shown to control the selectivity of reduced products, providing an additional route for improving reductive catalysis with metal oxides. In this work, we present kinetic resolution of the rate determining proton-transfer step of PCET to a titanium-doped POV, TiV_5_O_6_(OCH_3_)_13_ with 9,10-dihydrophenazine by monitoring the loss of the cationic radical intermediate using stopped-flow analysis. For this reductant, a 5-fold enhanced rate (*k*_PT_ = 1.2 × 10^4^ M^−1^ s^−1^) is accredited to a halved activation barrier in comparison to the homometallic analogue, [V_6_O_7_(OCH_3_)_12_]^1−^. By switching to hydrazobenzene as a reductant, a substrate where the electron transfer component of the PCET is thermodynamically unfavorable (Δ*G*_ET_ = +11 kcal mol^−1^), the mechanism is found to be altered to a concerted PCET mechanism. Despite the similar mechanisms and driving forces for TiV_5_O_6_(OCH_3_)_13_ and [V_6_O_7_(OCH_3_)_12_]^1−^, the rate of PCET is accellerated by 3-orders of magnitude (*k*_PCET_ = 0.3 M^−1^ s^−1^) by the presence of the Ti(iv) ion. Possible origins of the accelleration are considered, including the possibility of strong electronic coupling interactions between TiV_5_O_6_(OCH_3_)_13_ with hydrazobenzene. Overall, these results offer insight into the governing factors that control the mechanism of PCET in metal oxide systems.

## Abstract 2

The mechanism of proton-coupled electron transfer at the surface of titanium-substituted polyoxovanadate-alkoxide clusters can be tuned by judicious selection of substrate.

## Graphical abstract

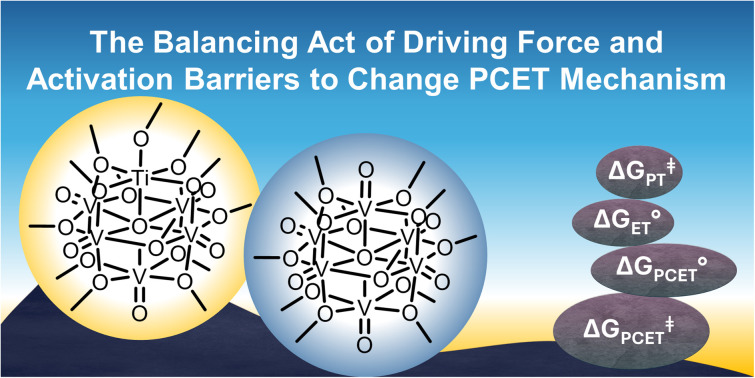

## Introduction

**Fig. 1:**
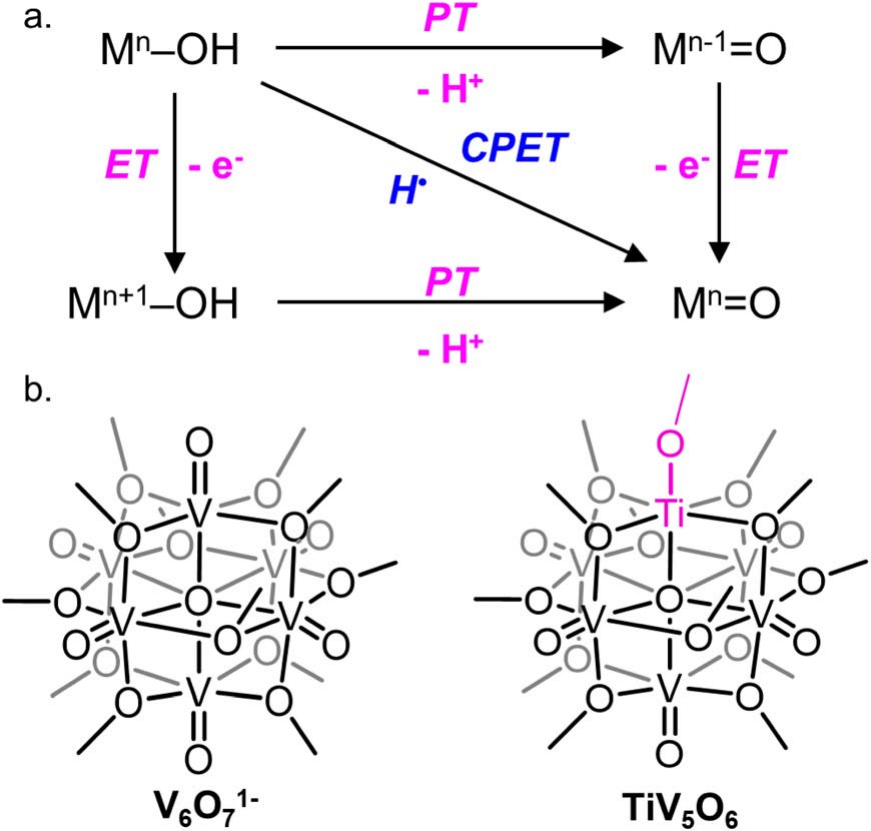
(a) Square scheme of PCET of a generic metal oxide and (b) cluster complexes studied in this work.

A central reaction invoked in the conversion of small molecules into commodity substrates is the transfer of proton and electron equivalents (*i.e.* H^+^/e^−^, H˙).^1^ Transfer of proton–electron pairs is described broadly as proton-coupled electron transfer (PCET) which can occur *via* either stepwise (electron or proton transfer, ET, PT) or concerted mechanisms (concerted proton–electron transfer, CPET; Fig. 1a).^2^ For small molecule activation, manipulation of the PCET mechanism has been shown to alter product distribution by avoiding unwanted side reactions.^3,4^ In this capacity, control of the delivery of H-atom equivalents is key for improving product selectivity.^5^

Heterogeneous catalysts, such as transition metal oxides, are promising materials for small molecule activation reactions involving PCET.^6,7^ The reactivity of metal oxides is dictated by the thermochemistry of the bound H-atom (H˙), presenting as aquo- or hydroxo-ligands, and are quantified by their bond dissociation free energies (BDFEs). In recent years, strides have been made toward understanding the structure–function relationship of the BDFEs, and has resulted in a library of metal oxides capable of (de)hydrogenation of small molecules containing E–H bonds (*e.g*. E = C, N, O).^1,8–10^ Knowledge of the thermodynamic properties of these materials allows for the opportunity to tune product selectivity and distribution, though less is known about the pathway of metal oxide mediated PCET.^11,12^ This is a result of the challenges that arise when investigating the mechanism of PCET on metal oxides due to inefficient methods available to researchers that allow monitoring of potential intermediates, and has resulted in some speculation in the process of the H-atom transfer on extended surfaces.^1,8,13^

To circumvent the challenges of studying bulk materials, our research team has turned to polyoxovanadate (POV) clusters as model systems for PCET on metal oxide surfaces (Fig. 1b).^14^ In these multi-metallic assemblies, the arrangement of bridging and terminal oxo sites oxides broadly mimics the surface of metal oxides, rendering them valuable for comparison to colloidal metal oxide nanoparticles.^15–17^ In addition, these POVs possess Robin and Day Class II delocalized electronic structures, mirroring the electronic structure of nanoscopic and bulk reducible metal oxides with the added benefit of a distinct chemical structure and solubility in organic solvent.^18,19^ Our group has investigated H-atom uptake for a series of these clusters, with the goal of understanding the structure–function relationship between synthetic modifications of the POV with thermodynamic and kinetic parameters for PCET.^20–24^ We have shown that terminal vanadyl (V^V^=O) sites can accept two H-atom equivalents resulting in a formal oxygen atom (O-atom) vacancy (V^III^–OH_2_), whereas POV containing bridging oxides (2V^V^–O) prefer to undergo these 2H^+^/2e^−^ transfers at disparate bridging oxo sites (2V^IV^–OH).^20,21,23^ We find that both nucleophilic sites undergo a rate limiting concerted H-atom transfer step, where the thermodynamics of O–H bond formation are dictated by the oxidation state distribution of vanadium ions composing the Lindqvist core.

**Scheme 1:**
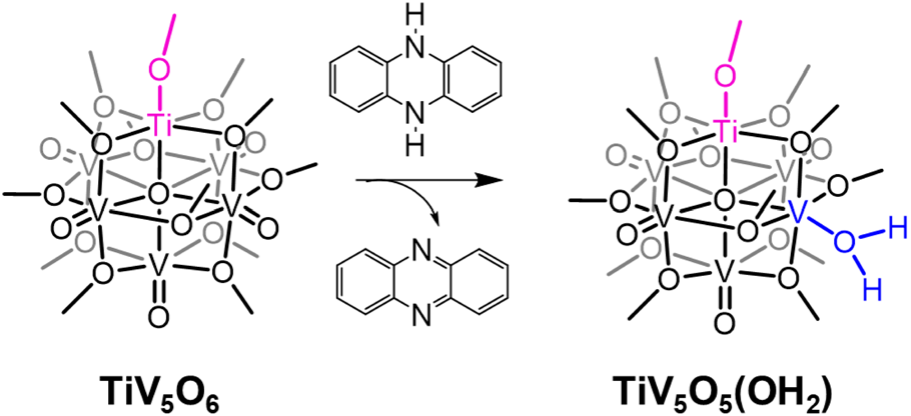
Net 2 H-atom reactivity of TiV_5_O_6_ with H_2_Phen.

Interested in expanding our knowledge of thermodynamic influences by synthetically altering POV clusters, we have recently turned our attention to the study of H-atom uptake in heterometal substituted POV assemblies. Previous work from our laboratory described O-atom defect formation to generate TiV_5_O_5_(OH_2_)(OCH_3_)_13_, TiV_5_O_5_(OH_2_), *via* PCET to a titanium-doped POV cluster, TiV_5_O_6_(OCH_3_)_13_, TiV_5_O_6_, using 9,10-dihydrophenazine (H_2_Phen) as a source of H-atoms (Scheme 1).^25^ The incorporation of a Ti^IV^ dopant into the Lindqvist assembly results in a change in the mechanism of H-atom uptake at the cluster surface; in the case of the all-vanadium POV cluster V_6_O_7_(OCH_3_)_12_^1−^ (V_6_O_7_^1−^), the reaction proceeds *via* concerted proton–electron transfer (CPET), whereas H-atom uptake at the titanium-doped assembly occurs through an electron transfer–proton transfer (ET–PT) mechanism. This shift in mechanism is attributed to an increase in driving force for ET 
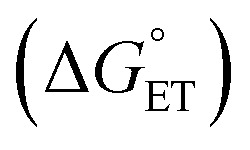
 from H_2_Phen upon cation substitution within the Lindqvist core. Indeed, inspection of the cyclic voltammogram (CV) of TiV_5_O_6_ in MeCN, shows that the most reducing V^V/IV^ couple (TiV_5_O_6_ + e^−^ → TiV_5_O_6_^1−^) is anodically shifted in comparison to the relevant V^V/IV^ couple of V_6_O_7_^1−^ (V_6_O_7_^1−^ + e^−^ → V_6_O_7_^2−^); this change in redox potential renders ET from H_2_Phen to TiV_5_O_6_ thermodynamically accessible 
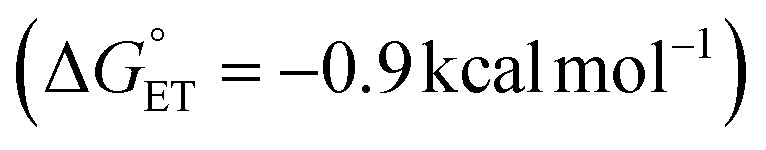
, whereas ET from H_2_Phen to V_6_O_7_^1−^ is endergonic 
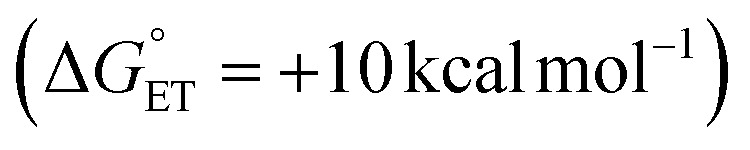
.^26^

Herein, we report the resolved kinetic data for the rate limiting proton-transfer (PT_limiting_), which follows an ET–PT_limiting_ mechanism from the reaction of TiV_5_O_6_ with H_2_Phen. Acceleration of the PT_limiting_, in comparison to rate-determining CPET reactivity for V_6_O_7_^1−^, is found to be dictated by lowering the activation barrier such that the stepwise reaction for TiV_5_O_6_ is 5-orders of magnitude faster than V_6_O_7_^1−^. Additionally, the stepwise reaction can be “turned off”, in favour of a concerted pathway for TiV_5_O_6_, by switching to a substrate that is a less potent reductant. Ultimately, this study provides context for changing the mechanism of PCET on metal oxide surfaces through modulation of elementary driving forces 
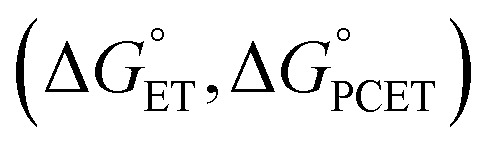
.

## Results and discussion

### Kinetic parameters for rate-limiting proton-transfer to a titanium-doped POV

**Fig. 2:**
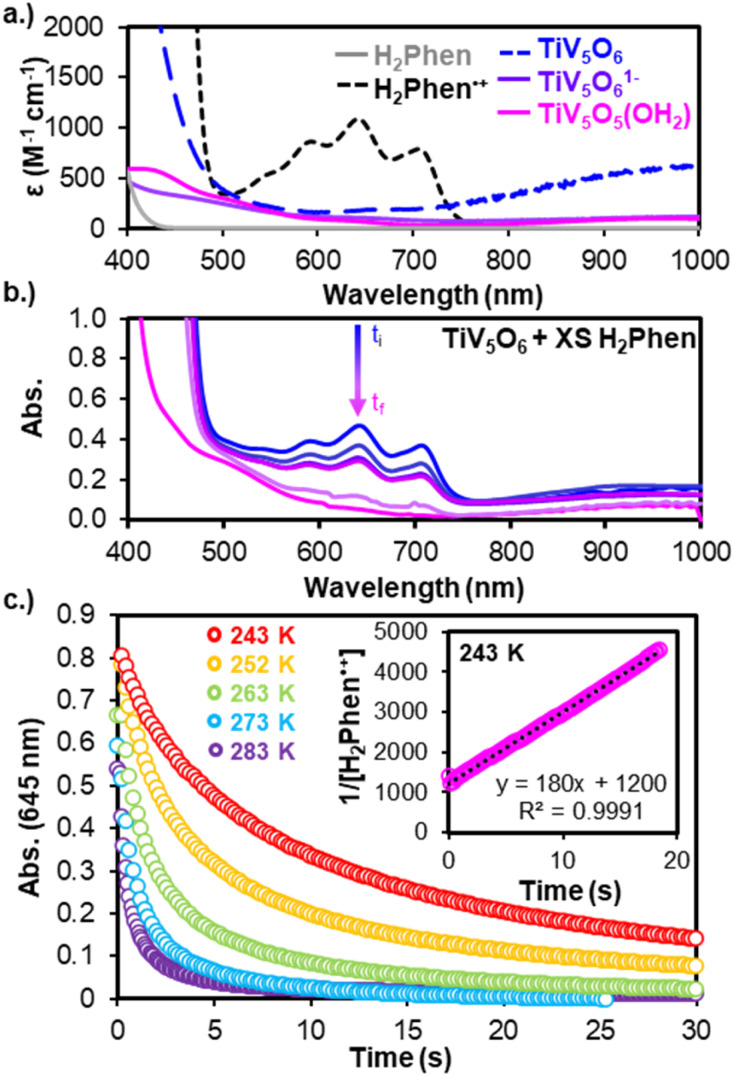
(a) Comparison of the EAS spectra of relevant species in MeCN. (b) TiV_5_O_6_ (0.75 mM) + H_2_Phen (7.5 mM) over the reaction coordinate at 243 K in MeCN. (c) Kinetic traces monitoring the loss of radical intermediate (H_2_Phen˙^+^, 0.75 mM) by PT to TiV_5_O_6_^1−^ (0.75 mM) over various temperatures (243–283 K) in MeCN. Inset shows second order relationship between [H_2_Phen˙^+^]^−1^ and time. The slope of the line is the second order rate constant, 180 ± 20 M^−1^ s^−1^ at 243 K.

In our initial report, evidence for the ET–PT mechanism was obtained from low temperature analysis of reaction mixtures by electronic absorption spectroscopy (EAS; Fig. 2). Following addition of substrate, immediate formation of an intermediate is observed, identified as H_2_Phen˙^+^ (Fig. 2a).^27^ Despite attempts to slow the reaction rate (*e.g*. lowered temperature, change in solvent), we were unable to fully resolve the kinetics of the ET–PT mechanism. Considering this, we turned to stopped flow analysis to experimentally determine the rates of H-atom uptake at TiV_5_O_6_.

In prior reports describing H-atom uptake at the surface of POV clusters, reaction progress has been monitored by measuring the change in absorption at the V^V/IV^ intervalence charge transfer (IVCT; V^IV^ → V^V^) band in the near-infrared region of the spectrum (*λ* = 1000 nm).^21,24,28^ Following the addition of H_2_Phen to TiV_5_O_6_ (Ti^IV^V^V^V_5_^IV^), the IVCT absorption feature is quenched (<0.6 s). We assign the loss of this feature to the reduction of the neutral TiV_5_O_6_ cluster to the mono-anionic assembly, TiV_5_O_6_^1−^ (Ti^IV^V_5_^IV^), which occurs during the initial ET step of H-atom uptake (Fig. 2a). The rapid ET is consistent with fast heterogeneous electron transfer reported for the first V^V/IV^ couple of TiV_5_O_6_ (*k*_ET_ = 3.1 × 10^−2^ cm^−1^ s^−1^).^26^

We shifted our attention to monitoring the kinetics of the rate-limiting PT step by following the loss of the absorption feature corresponding to the intermediate, dihydrophenazinium, H_2_Phen˙^+^ (*λ* = 645 nm; Fig. 2b).^27^ Experiments reveal that the consumption of H_2_Phen˙^+^ over the reaction coordinate has no dependency on the initial concentration of H_2_Phen (Fig. S1†). This observation can be justified by the anticipated rate expression for the rate-limiting PT step being first order in reduced cluster and first order in H_2_Phen˙^+^. The concentration of H_2_Phen˙^+^ is determined by the initial ET step and limited under the studied reaction conditions by the concentration of TiV_5_O_6_ (see Experimental section for more details). In this experiment, it is likely that quantitative ET occurs between H_2_Phen and TiV_5_O_6_ (*E*^ox^_1/2_ = −0.33 V *vs.* Fc^+/0^ and *E*^red^_1/2_ = −0.29 V *vs.* Fc^+/0^, for H_2_Phen and TiV_5_O_6_, respectively; 
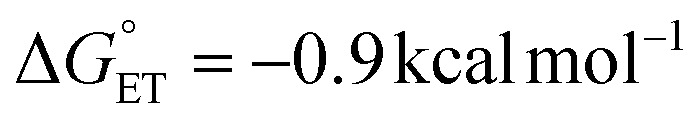
), resulting in the formation of equimolar amounts of H_2_Phen˙^+^ and TiV_5_O_6_^1−^. Second order fitting of the kinetic data can be applied, resulting in a plot of [H_2_Phen˙^+^]^−1^*vs.* time with a linear relationship to three half-lives (Fig. 2c). The second order rate constant (*k*_PT_) can be determined from the slope of the line, *k*_PT_ = 180 ± 20 M^−1^ s^−1^ at 243 K. We note that to resolve the rate of PT, kinetic experiments were conducted at lower temperatures.

The preferred mechanism of PCET is dictated by reaction pathways with the lowest energy barriers. The origin of CPET is due to the preference to couple the ET and PT reactions; in a stepwise mechanism the electron and proton are transferred in two steps, resulting in the formation of charged (*i.e.* high-energy) intermediates that are often credited with slowing the overall rate of the net H-atom transfer reaction.^29–32^ However, in the case of TiV_5_O_6_, a substantial acceleration of the rate of H-atom uptake is observed in comparison to its homometallic analogue as a consequence of switching to a stepwise mechanism. The experimentally derived second order rate constant (*k*_exp_) for H-atom transfer from H_2_Phen to V_6_O_7_^1−^ was reported to be 5.1 ± 0.4 M^−1^ s^−1^ at 298 K in MeCN.^33^ Comparison to TiV_5_O_6_ reveals that even at reduced temperatures (298 K *vs.* 243 K), the rate determining step is an order of magnitude faster than V_6_O_7_^1−^.

**Fig. 3:**
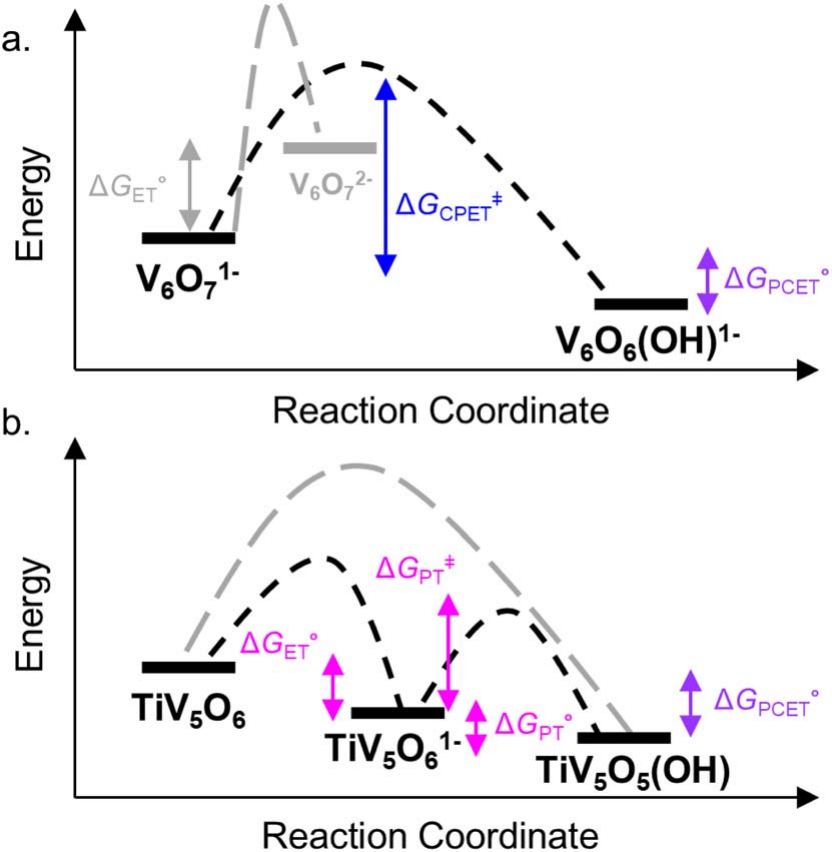
Reaction coordinate diagram for reactivity of V_6_O_7_^1−^ (top, a) and TiV_5_O_6_ (bottom, b) with H_2_Phen. Activation energy for the CPET process is shown in blue traces. ET–PT free energy changes and PT activation energy are shown in pink traces. Free energy for PCET process is shown in purple. Grey traces show pathways that are not favourable. All values are listed in Table 1.

Reaction rates of PCET are proposed to be dependent on a multitude of factors such as driving forces of PT and ET 
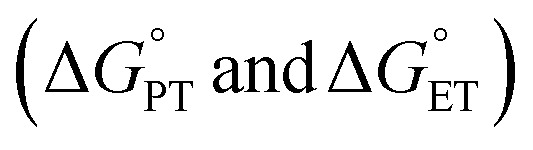
, reorganization energy, as well as tunnelling distances. For example, increasing the driving forces of 
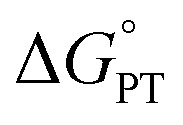
 and 
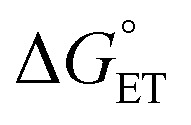
 may provide a route to achieve enhanced reaction rates.^34,35^ Considering this, we next examined the driving forces for the rate determining step for both TiV_5_O_6_ (PT) and V_6_O_7_^1−^ (CPET) to assess the origin of the acceleration of reaction rates observed. Using the experimentally derived E_1/2_ and BDFE(E–H)_avg_, (E = O, N, respectively) values of TiV_5_O_6_ and H_2_Phen, the Bordwell equation can be used to approximate the p*K*_a_ (p*K*^approx.^_a_) of each substrate and obtain 
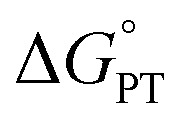
 (see Experimental for more details). For TiV_5_O_6_, both 
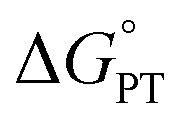
 and 
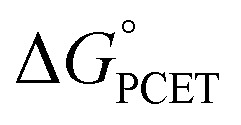
 are both small (Table 1 and Fig. 3). This finding is surprising, given that the rate is substantially accelerated for the stepwise pathway. For this reason, activation barriers are valuable when contemplating the origins of the acceleration of PCET.

**Table 1:** Thermodynamic and kinetic values describing reactivity of homo- and hetero-metallic POV with H_2_Phen

	bV_6_O_7_^1−^	cTiV_5_O_6_
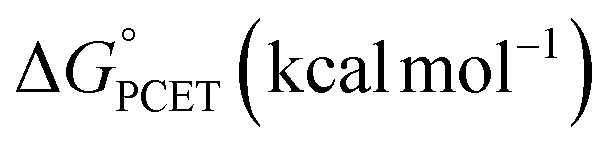	−0.7^a^	−0.9^a^
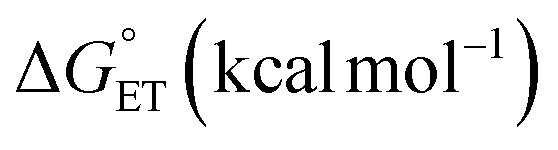	+10^a^	−0.9^a^
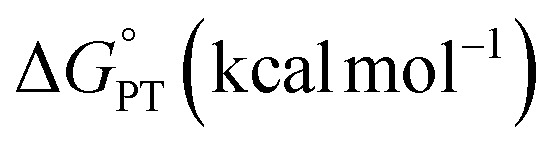	−2	−3 × 10^−2^
*k*_exp_ @ 298 K (M^−1^ s^−1^)	4.5 × 10^−1^^a^	1.2 × 10^4^
KIE (*k*_H_/*k*_D_)	1.7 ± 0.2^a^	4.6 ± 0.2
Δ*H*^‡^ (kcal mol^−1^)	7 ± 1^a^	11 ± 2
Δ*S*^‡^ (cal mol^−1^ K^−1^)	−37 ± 2^a^	−4 ± 4
Δ*G*^‡^ 298 K (kcal mol^−1^)	18 ± 1^a^	10 ± 2

a Determined in referenced work.^23,25^

b CPET kinetic parameters.

c ET–PT_limiting_ kinetic parameters.

**Fig. 4:**
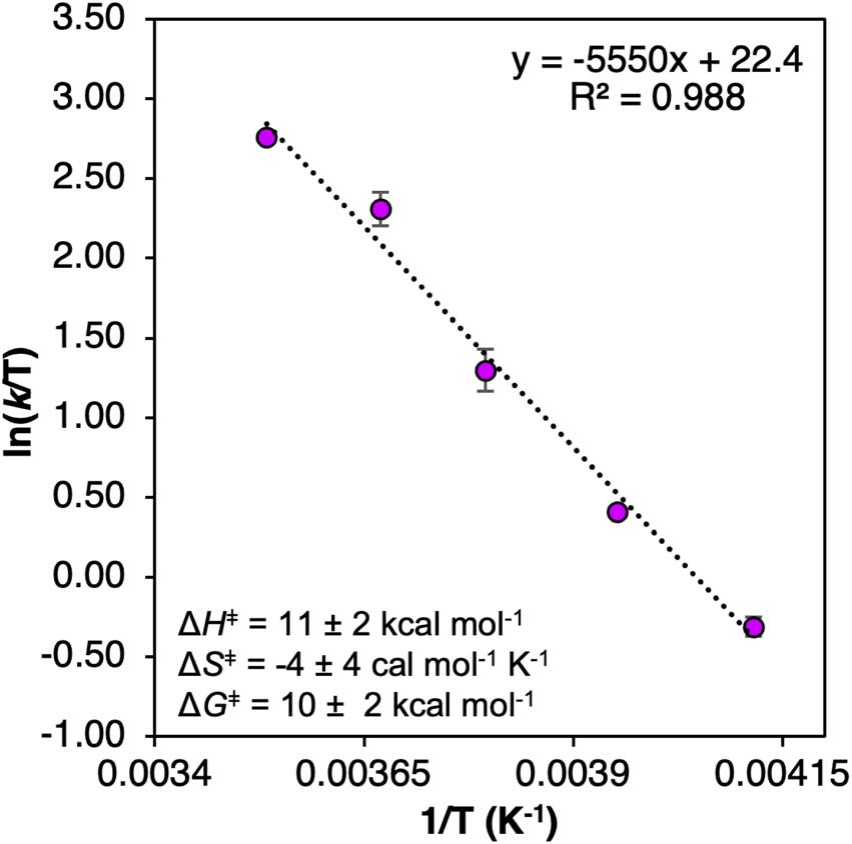
Eyring plot of the PT from TiV_5_O_6_^1−^ and H_2_Phen˙^+^ 243–283 K in MeCN. Inset shows activation parameters. Error bars for each data point are determined from the standard deviation between three trials.

We next examined the activation parameters of proton uptake *via* Eyring analysis for TiV_5_O_6_ to further evaluate kinetic factors for the disparate mechanisms. The activation parameters of the transition state of the PT_limiting_ step of H-atom uptake at TiV_5_O_6_ were obtained through variable-temperature kinetic analysis. Using experimental conditions described above, *k*_PT_ values were determined at temperatures ranging from 243 to 283 K (Fig. 2c, 4 and S2†). Enthalpy and entropy of activation (Δ*H*^‡^ and Δ*S*^‡^) of PT from H_2_Phen˙^+^ to TiV_5_O_6_^1−^ are determined to be 11 ± 2 kcal mol^−1^ and −4 ± 4 cal mol^−1^ K^−1^, respectively. Using the experimentally determined entropy and enthalpy of activation, the free energy of activation (Δ*G*^‡^) for proton transfer was determined to be 10 ± 2 kcal mol^−1^ at 298 K. Δ*G*^‡^ for TiV_5_O_6_ is ∼8 kcal mol^−1^ smaller than Δ*G*^‡^ for CPET to V_6_O_7_^1−^, Δ*G*^‡^_CPET_ (Table 1). The reduction in Δ*G*^‡^ results in substantial discrepancies in rates between TiV_5_O_6_ and the homometallic analogue and can be accredited to the lower intrinsic barriers for the stepwise ET and PT steps of the mechanism. In addition, the energy of the intermediate is expected to scale with the barrier height, and the transition from the charged intermediate to a neutral cluster likely aids in minimizing the activation barrier. For TiV_5_O_6_, the overall acceleration of the PCET reactivity is imparted by smaller activation energies (Δ*G*^‡^_ET_ and Δ*G*^‡^_PT_) for the stepwise mechanism in comparison to CPET (Δ*G*^‡^_CPET_).^36^

Thermochemical parameters describing the formation of the transition state of the rate-determining step of a reaction are frequently used to distinguish between mechanisms of PCET to metal oxo complexes.^37–41^ Generally, a large and negative value for Δ*S*^‡^ is indicative of formation of an ordered, hydrogen-bonding donor/acceptor pair in the activated state. This, coupled with a Δ*H*^‡^ value that is small and positive has been invoked in CPET mechanisms. Exemplary of this are the activation parameters listed in Table 1 for the reactivity of V_6_O_7_^1−^ with H_2_Phen. Considering the established activation parameters for CPET type mechanisms, deviations from these “ideal” values are suggested as a justification when the mechanism is suspected to change. For example, Borovik and coworkers observe a mechanism change from CPET (Δ*H*^‡^ = 5 kcal mol^−1^, Δ*S*^‡^ = −49 cal mol^−1^ K^−1^, Δ*G*^‡^ = 19 kcal mol^−1^) to PT–ET (Δ*H*^‡^ = 14 kcal mol^−1^, Δ*S*^‡^ = −14 cal mol^−1^ K^−1^, Δ*G*^‡^ = 18 kcal mol^−1^) upon increasing the basicity of Mn^III^ oxo complex.^42^ This mechanism assignment is supported by a more positive value of Δ*S*^‡^ for the PT_limited_ process, which the authors attribute to solvation of the ion pair following PT (*e.g*. Mn complex and anion substrate). In our system, there is a small, negative change in disorder in the generation of the transition state for PT from H_2_Phen˙^+^ to TiV_5_O_6_^1−^. This Δ*S*^‡^ can be rationalized by changes in solvation that are associated with the transition state. In a polar solvent like MeCN, the solvent is expected to arrange around the charged complex, [TiV_5_O_6_^1−^][H_2_Phen˙^+^]. To achieve the activated complex for proton transfer, changes in charge allocation must occur. The ionic complex in MeCN must rearrange such that there is a new neutral species, which results in reordering of the solvation shell with arranged dipole moments, explaining the small magnitude of Δ*S*^‡^.

Kinetic analysis of the addition of deuterated 9,10 dihydrophenazine-*d*_2_ (D_2_Phen) to TiV_5_O_6_ reveals a kinetic isotope effect (KIE) for the ET–PT_limiting_ mechanism. The KIE is obtained by extrapolating the rate at room temperature from activation parameters for PCET to TiV_5_O_6_, which are summarized in Table 1. Upon isotope labelling of substrate (98% ^2^H as determined by ^1^H NMR spectroscopy), the rate of PT is slowed (*k*^D^_PT_ = 2600 M^−1^ s^−1^, Fig. S3†), resulting in a KIE of 4.6 ± 0.2. This value supports movement of a proton during the rate determining step of the reaction, in good agreement with the proposed PT-limited mechanism. Notably, the KIE observed in H-atom uptake at the Ti-doped POV cluster is larger than that reported for V_6_O_7_^1−^ (KIE = 1.7 ± 0.2). While it is possible that this is a result of more extensive tunnelling of the proton in the ET–PT mechanism of the PCET to TiV_5_O_6_, we instead propose that the increase in KIE value measured for TiV_5_O_6_ is due a small driving force for PT. Thermoneutral proton transfers (Δ*G*° ≈ 0) have been shown to exhibit larger KIEs.^43,44^ Indeed, the 
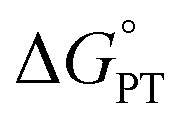
 value is found to be small (Table 1).

### Computational insights into free energy changes for PCET to POV with H_2_Phen

**Fig. 5:**
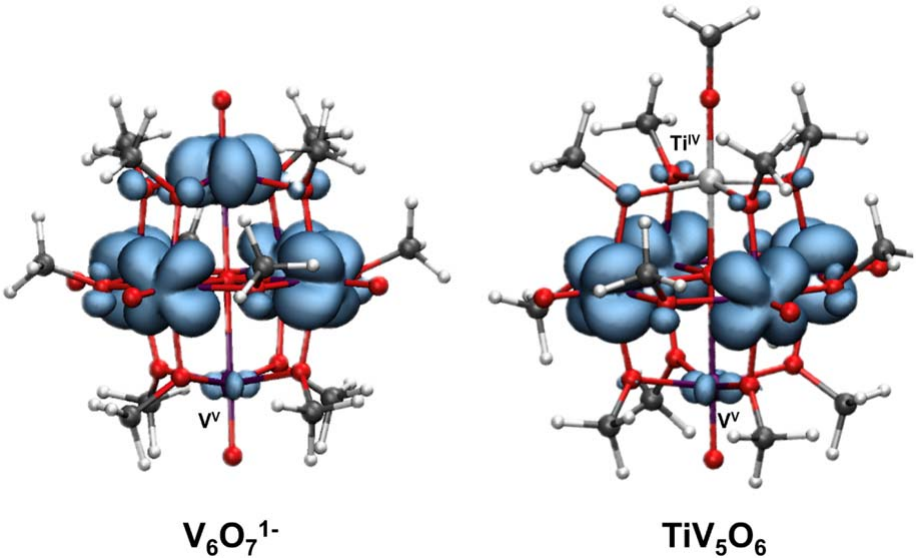
Spin density in the pristine all-vanadium V_6_O_7_^1−^ (left) and TiV_5_O_6_ (right). Isosurface 0.003. Colour code: vanadium in purple, titanium in light grey, oxygen in red, carbon in dark grey, and hydrogen in white.

Density functional theory (DFT) calculations were performed on both V_6_O_7_^1−^ and TiV_5_O_6_ to provide insight into consequences of dopant installation on the electronic structure of the assembly. The goal of the calculations is to aid in the elucidation of factors that contribute to the different mechanisms between the two assemblies. For both V_6_O_7_^1−^ and TiV_5_O_6_, spin density maps reveal that the V^V^ center is partially localized, which is in agreement with the assignment of the mixed-valent clusters as Robin and Day Class II systems (Fig. 5).^19,45^ In the case of V_6_O_7_^1−^, the partial localization is induced by the methoxy bridging ligands breaking the ideal Lindqvist O_h_ core symmetry, whereas the symmetry of TiV_5_O_6_ is further broken by the presence of the Ti^IV^ ion.

Computational support to the assignment of mechanisms of PCET to the homo- and hetero-metallic POV clusters with H_2_Phen was obtained through DFT calculations. It is important to note that the computational data presented in this work are strictly thermodynamic values and do not consider kinetic values (*i.e*. activation barriers), vital for explicit mechanistic assignment. Activation barriers associated with ET, PT, and CPET processes are essential in establishing mechanism; however, obtaining these values in open shell multimetallic systems like POVs is nontrivial. Nevertheless, we believe that the thermodynamic data holds merit in conjunction with the experimentally derived kinetic data to propose the most likely pathways of PCET in the presented POVs.

**Fig. 6:**
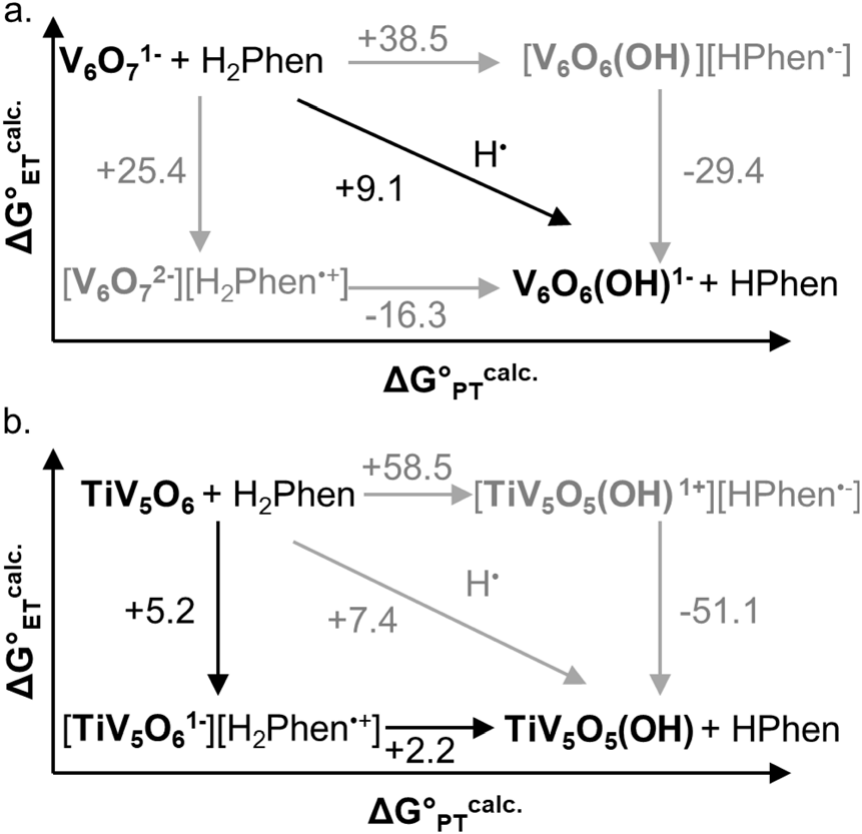
Computationally derived thermochemistry of V_6_O_7_^1−^ (top, a) and TiV_5_O_6_ species (bottom, b) with H_2_Phen. Horizontal arrows are associated with PT free energies, vertical arrows with ET free energies, and diagonal arrows with CPET free energies. The black arrows represent the most favourable path from V^V^=O to V^IV^–OH. All presented energies are Gibbs free energies in kcal mol^−1^.

Gibbs free energy differences are determined by optimization of the geometries of the reactants and products of the elementary PCET steps (*i.e.* PT, ET, CPET) in the gas phase. The values presented in Fig. 6 are representative of the previously proposed rate-determining step, which is suggested to be the formation of the single H-atom transfer product containing V(iv)–OH. This causes the first net H-atom transfer to be slightly uphill, as demonstrated by the positive values reported in Fig. 6. However, the overall reactions are the transfer of two H-atoms (*i.e.* 2e^−^ + 2H^+^) and remain downhill. In agreement with the experimentally determined mechanism, calculations suggest that there is thermodynamic preference for CPET to V_6_O_7_^1−^ (V^V^=O → V^IV^–OH, Fig. 6a). This is because the off diagonal mechanisms (*e.g.* Δ*G*^calc.^_ET_ = +25.4 kcal mol^−1^, Δ*G*^calc.^_PT_ = + 38.5 kcal mol^−1^) are much higher in energy due to the formation of ion paired intermediates.

Considering the thermodynamic landscape provided by the computational analysis in this section, H-atom uptake at TiV_5_O_6_ following addition of H_2_Phen occurs *via* a sequential ET–PT mechanism (Fig. 6b).^46^ Thermodynamically, the ET is favored by 2.2 kcal mol^−1^ over CPET, which is followed by PT to generate the V^IV^–OH. In the previous section, experimental data suggested that the PT is the rate-determining step, which is in good agreement with the calculated thermodynamic data presented here. We note that the thermodynamic values displayed in Fig. 6 show a greater change in free energy (+25.4 kcal mol^−1^) than the experimental values listed in Table 1 (+10 kcal mol^−1^). We attribute this discrepancy in free energy changes due to the complexity of the electronic structures of the POV clusters investigated. Nevertheless, we find the qualitative agreement between the experimental and computational thermodynamic schemes as satisfactory.

The origin of the observed stepwise ET–PT for the TiV_5_O_6_ over a CPET mechanism can be explained by differences in the energetics of the frontier molecular orbitals. The LUMO in the titanium doped species is significantly lower in energy thus favoring an initial ET step over CPET (−2.53 eV for V_6_O_7_^1−^ and −3.47 eV for TiV_5_O_6_). As noted by Hammes-Schiffer and co-workers, the use of isodesmic reactions benefit from error cancellation inherent to density functional theory and implicit solvation models.^47^ In this aspect, we observed a stronger DFT functional dependence in the non-concerted ET–PT over the CPET, likely due the different description of the intermediate V^IV^=O by different exchange–correlation functionals.^48^ For example, PBE0 functional suggests more favorable thermodynamic values along the ET–PT pathway for TiV_5_O_6_, in agreement with the experimental results, while M06 reveals a CPET as the most downhill path (Fig. S4†). The thermodynamic values for PCET to V_6_O_7_^1−^ are independent on the DFT functional for the V^V^=O to V^IV^–OH transformation, where M06 and PBE0 predict CPET as the energetically favorable route (Fig. S5†). Ultimately, in regimes where the driving forces for stepwise and concerted pathways are comparable, computational analysis must be interpreted cautiously while paying mind to experimental kinetic data, as the DFT for POVs in this work cannot account for activation barriers.

### Kinetic investigation with a mild H-atom donor and POV clusters

**Scheme 2:**
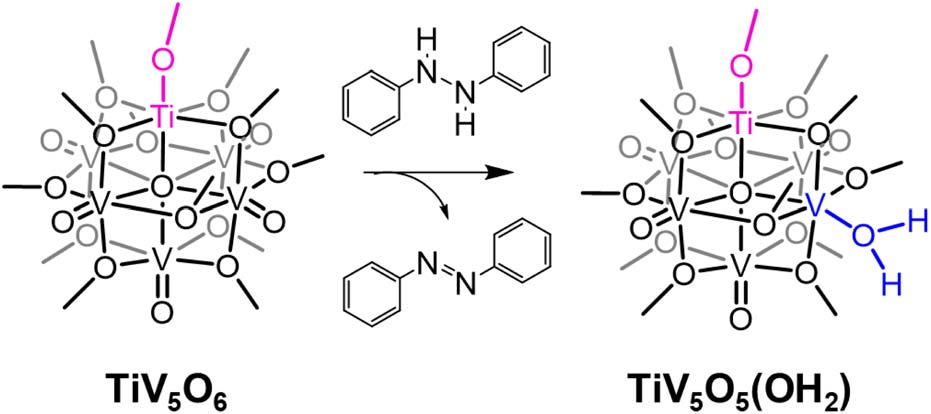
Reactivity of TiV_5_O_6_ with H_2_Azo.

Experimental and computational evidence discussed in this work suggest that sequential delivery of the proton/electron pair *via* an ET–PT mechanism to TiV_5_O_6_ is a consequence of the exergonic electron transfer step from H_2_Phen to the cluster. We next became interested in expanding the substrate scope to probe the extent of the influence 
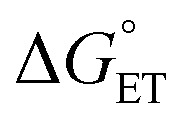
 has on Δ*G*^‡^, which will alter the mechanism. Accordingly, we aimed to modulate the system to balance 
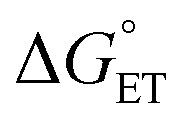
 between TiV_5_O_6_ and substrate such that PCET can be facilitated *via* a CPET mechanism. To accomplish this, we investigated PCET from hydrazobenzene (H_2_Azo) to TiV_5_O_6_ (Scheme 2). For H_2_Azo, the H-atom is similarly sourced from a weak N–H bond (BDFE(N–H)^MeCN^_avg_ = 60.7 kcal mol^−1^)^2^ and the oxidation potential is less accessible to the Ti-doped POV cluster (
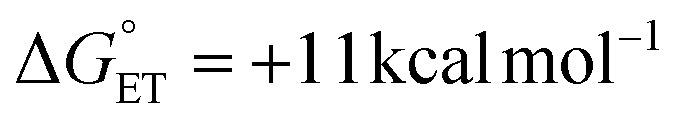
, Fig. S6†). Analysis of the products *via*^1^H NMR spectroscopy of the crude reaction mixture of TiV_5_O_6_ with H_2_Azo indicates formation of TiV_5_O_5_(OH_2_) (Fig. S7†), as well as the expected dehydrogenated organic substrate azobenzene (Azo).

**Fig. 7:**
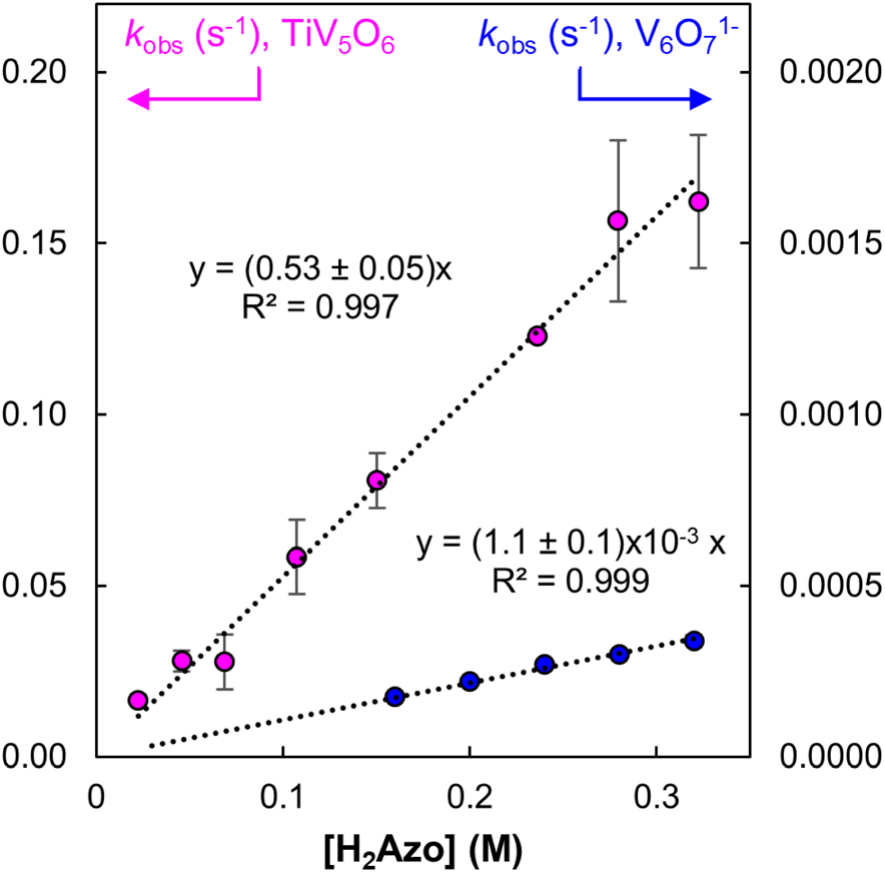
Plot of *k*_obs_ as a function of [H_2_Azo] for TiV_5_O_6_ (pink circles, left *Y*-axis) and V_6_O_7_^1−^ (blue circles, right *Y*-axis) at 318 K. Concentration of POV were held constant at 0.75 mM, and reductant concentration was varied. The slope of the resultant lines provides the experimentally derived second-order rate constants, *k*_exp_. Error bars for each data point are determined from the standard error of triplicate trials.

To probe the mechanism of PCET from H_2_Azo to TiV_5_O_6_, kinetic analyses were performed using EAS (Fig. S8†). Comparison of the averages of the BDFE's of the complexes suggest a 
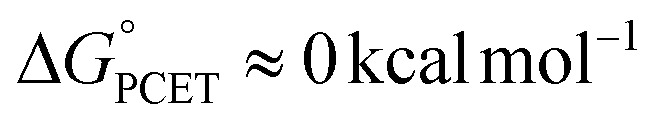
, requiring the addition of excess substrate to drive the reaction to completion. Kinetic analyses of PCET between TiV_5_O_6_ with H_2_Azo was achieved by monitoring reaction progression under pseudo first order reaction conditions at elevated temperature (318 K). Loss of the IVCT bands for the V^V/IV^ transition at 1050 nm allows for facile monitoring of the amount of parent cluster, TiV_5_O_6_. Kinetic traces at this wavelength can be fit to an exponential decay function using least squares regression method to obtain the pseudo-first order rate constant, *k*_obs_ (Fig. S9 and S10†). Varying the concentration of reductant results in a linear relationship with *k*_obs_ indicating a first-order dependence on [H_2_Azo] for reactivity with TiV_5_O_6_ (Fig. 7). Larger concentrations of reductant were probed for TiV_5_O_6_ to rule possible saturation kinetics and showed no levelling effect (Fig. S11†). We attribute the larger error at high concentration to be caused by increased uncertainty due to the faster reaction rates. From the slope of the line, the experimentally derived second order rate constant is obtained, *k*_exp_ = 0.53 ± 0.05 M^−1^ s^−1^ at 318 K. The observed rate of reaction with H_2_Azo is substantially slower in comparison to its reactivity with H_2_Phen (*k*_PT_ = 1.2 × 10^4^ M^−1^ s^−1^, 298 K). However, the rate is accelerated in comparison with the homometallic cluster, where H-atom transfer to V_6_O_7_^1−^ from H_2_Azo proceeds with *k*_exp_ = (1.1 ± 0.1) × 10^−3^ M^−1^ s^−1^ at 318 K (Fig. 7 and S12†).

To account for the different number of active sites on the POV clusters, we invoke a statistical factor (*n*) based on the two H-atoms transferred and number of reactive vanadyls to accurately compare rate constants.^33^ For V_6_O_7_^1−^, all six terminal V=O sites can accept an H-atom, whereas for TiV_5_O_6_, only the four equatorial V=O bonds are believed to participate in H-atom uptake reactions.^23^ After accounting for the probability factor associated with the reactive sites on each POV, *k*_PCET_ values can be determined. The second order rate constant for a rate-limiting H-atom transfer to each POV cluster at 318 K are *k*_PCET_ = 6.6 × 10^−2^ M^−1^ s^−1^ (TiV_5_O_6_) and 9.2 × 10^−5^ M^−1^ s^−1^ (V_6_O_7_^1−^). The heterometal-doped POV cluster, TiV_5_O_6_, reacts three orders of magnitude faster than the homometallic assembly. This acceleration of the HAT reaction is reminiscent of the previous example employing H_2_Phen as a reductant, where accelerated rates are a byproduct of the reduced Δ*G*^‡^ following an ET–PT mechanism. However, it is important to reiterate that ET from H_2_Azo to TiV_5_O_6_ is endergonic 
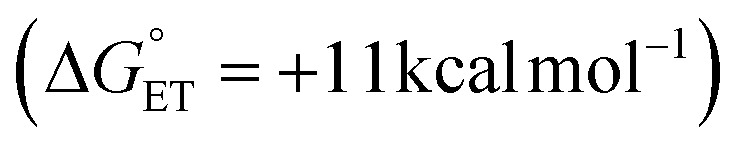
, thus making a purely ET–PT mechanism unfavourable.

We next performed kinetic analyses with deuterium-labelled substrate, D_2_Azo with the POVs, (99% ^2^H as determined by ^1^H NMR spectroscopy). Deuterium labelling of the substrate results in a KIE of 1.7 ± 0.2 for V_6_O_7_^1−^ (Fig. S13–S16†), whereas no KIE was observed for the titanium-doped cluster (KIE = 1.0 ± 0.2). The lack of a KIE suggests that the rate-limiting step for the reaction between TiV_5_O_6_ with H_2_Azo does not involve a proton. However, in rare examples, KIE values close to origin have been observed for CPET reactions.^29,43,49,50^ Hammarström and coworkers summarized the effects that could yield a KIE of ∼1 for CPET, which are briefly described as (1) proton transfer potential that are less harmonic than Morse potentials, and (2) proton tunnelling distances that are minimized by thermal distributions.^29^

**Fig. 8:**
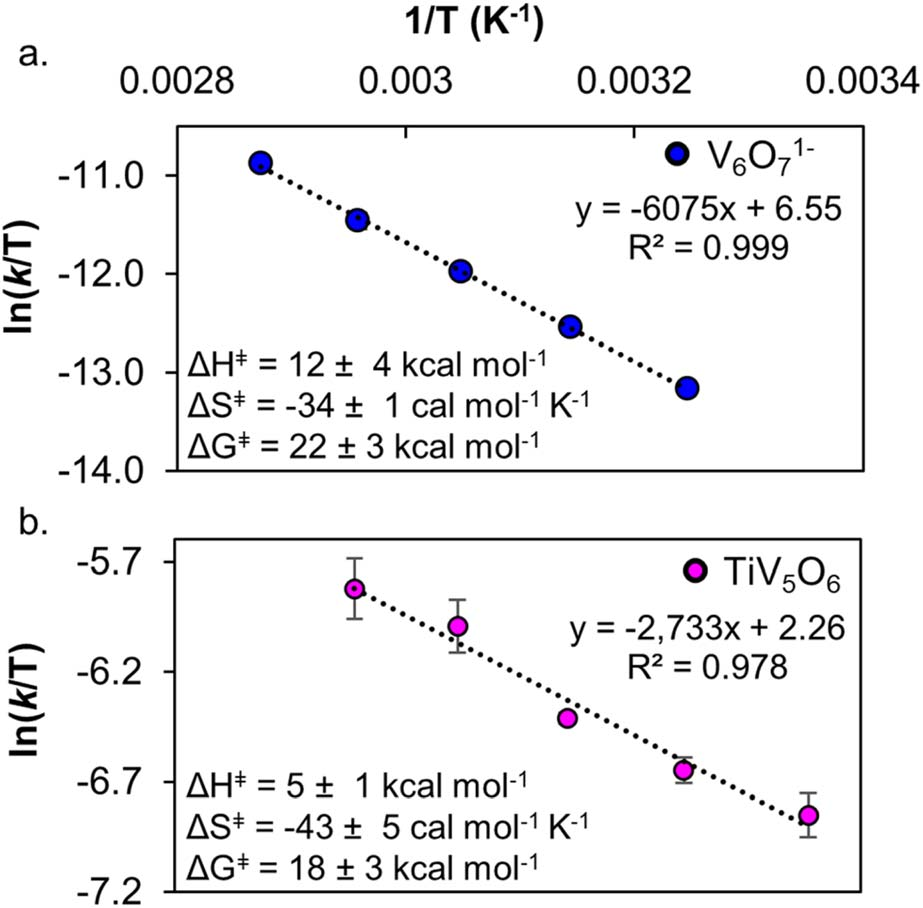
Eyring plot of PCET from H_2_Azo to V_6_O_7_^1−^ 298–338 K in MeCN (top, a). Eyring plot of PCET from H_2_Azo to TiV_5_O_6_ 308–348 K in MeCN (bottom, b). Inset shows activation parameters. Error bars for each data point are determined from the standard deviation between triplicate trials.

Temperature-dependent kinetic analysis was performed on the reactions of H_2_Azo with TiV_5_O_6_ and V_6_O_7_^1−^ to further probe the mechanism of PCET. As described earlier, from the slope and intercept of the line on the Eyring plot, the activation parameters for PCET to POV clusters can be determined (Fig. 8, S17 and S18,† see Experimental for more details). Interestingly, Δ*G*^‡^ for TiV_5_O_6_ (Δ*G*^‡^ = 18 ± 3 kcal mol^−1^) and V_6_O_7_^1−^ (Δ*G*^‡^ = 22 ± 3 kcal mol^−1^) are statistically equivalent (Table 2) at 298 K. Both clusters have activation parameters that are reminiscent to values reported previously for CPET reactions, with large and negative activation entropies and small activation enthalpies.^23,38,39,42,51^ We attribute the large and negative Δ*S*^‡^ to be caused by the need for the cluster and substrate to form an H-bonded pair in the rate-determining CPET. The activation parameters suggest that both POV clusters undergo H-atom uptake *via* a CPET mechanism with H_2_Azo.

**Table 2:** Kinetic and Thermodynamic parameters for reactivity of POV with H_2_Azo

	V_6_O_7_^1−^	TiV_5_O_6_
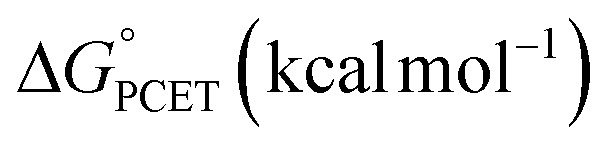	0.5	0.3
*k* @ 298 K (M^−1^ s^−1^)	3.0 × 10^−4^	3.0 × 10^−1^
Δ*H*^‡^ (kcal mol^−1^)	12 ± 1	5 ± 1
Δ*S*^‡^ (cal mol^−1^ K^−1^)	−34 ± 4	−43 ± 5
Δ*G*^‡ 298 K^ (kcal mol^−1^)	22 ± 3	18 ± 3

**Fig. 9:**
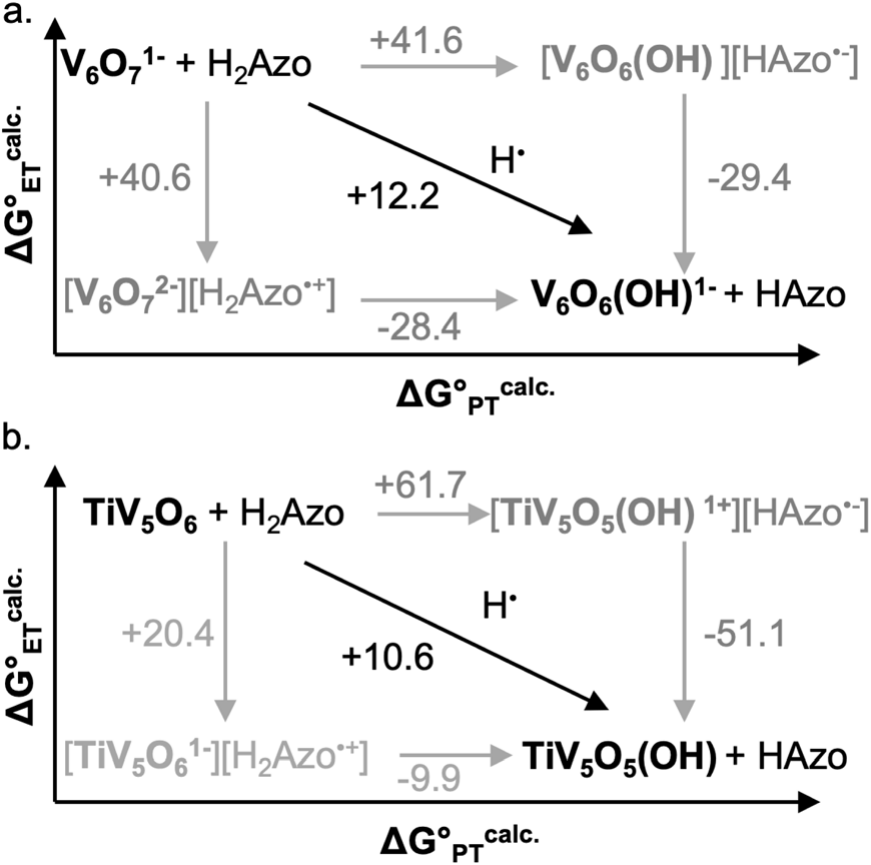
Computationally derived thermochemistry of V_6_O_7_^1−^ (top, a) and TiV_5_O_6_ species (bottom, b) with H_2_Azo. Horizontal arrows are associated with PT free energies, vertical arrows with ET free energies, and diagonal arrows with CPET free energies. The black arrows represent the most favourable path from V^V^=O to V^IV^–OH. All presented energies are Gibbs free energies in kcal mol^−1^.

Using the computational methods described above, we find that the use of H_2_Azo as a source of H-atom equivalents yields a thermodynamic landscape where the most likely mechanism is CPET for both V_6_O_7_^1−^ and TiV_5_O_6_ (Fig. 9). This is evident upon inspection of the stepwise mechanisms for TiV_5_O_6_, where the ET from H_2_Azo becomes unfavorable (Δ*G*^calc.^_ET_ = +20.4 kcal mol^−1^). A PT-led mechanism can also be ruled out; the mildly basic cluster is incapable of deprotonating H_2_Azo, as indicated by the highly endergonic PT determined *via* computations (Δ*G*^calc.^_PT_ = 61.7 kcal mol^−1^). We suggest that the thermodynamic theoretical data presented imply that transfer of an H-atom to TiV_5_O_6_ from H_2_Azo occurs by CPET.

Taken together, the data presented in this section suggests that the operative mechanism of H-atom uptake on TiV_5_O_6_ from H_2_Azo is CPET. We find that the mechanism of PCET of the Ti-doped cluster can be predicted and effectively controlled by modulating the free energy barriers for the elementary steps (*i.e.*
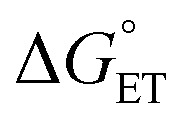
, 
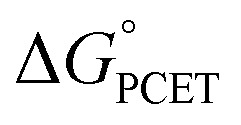
) of the H-atom transfer reagent. When 
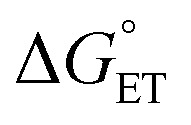
 is exergonic, an ET–PT pathway is favored, whereas when 
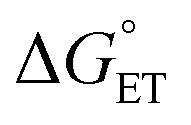
 becomes less favorable, a mechanism crossover is encountered to CPET.

With experimental support for a CPET mechanism, there remains the subject of the accelerated reaction rate observed for TiV_5_O_6_ and H_2_Azo in comparison to V_6_O_7_^1−^. It has been suggested that the rate of CPET may be enhanced by an “imbalanced” or “asynchronous” transition state, which describes the transition state of the H˙ transfer with the H^+^/e^−^ pair at inequal distances on the reaction coordinate. To measure the extent of the imbalance in the transition state, Srnec and coworkers proposed an asynchronicity factor (*η*) which is proportional to the free energy changes associated with the proton transfer 
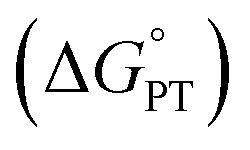
 and the electron transfer 
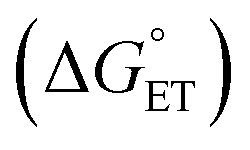
 between reduced and oxidized substrates (eqn (1)).^52^ This value is then approximated by the difference in free energy changes of PT and ET, 
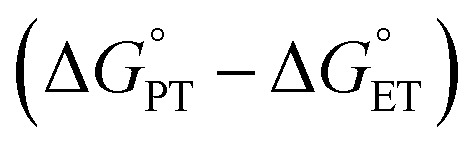
 where F is Faraday's constant.^53^1
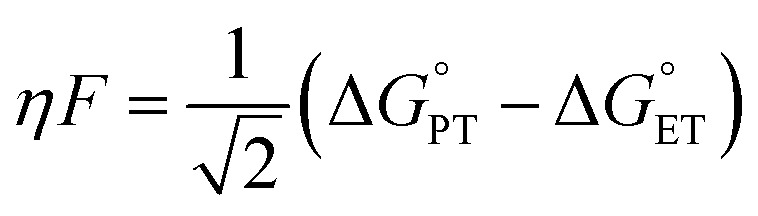


Thus, a larger 
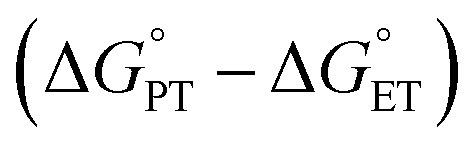
 would be indicative of more extensive imbalance. For the clusters presented in this work, the titanium-doped POV exhibits more modest changes to the redox potential and p*K*_a_ contrasted to V_6_O_7_^1−^ (see Table 1 for values), which results in only minor changes to the 
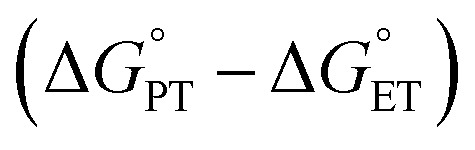
 value. This implies that asynchronicity would not play a substantial role in the acceleration of PCET to TiV_5_O_6_.

**Scheme 3:**
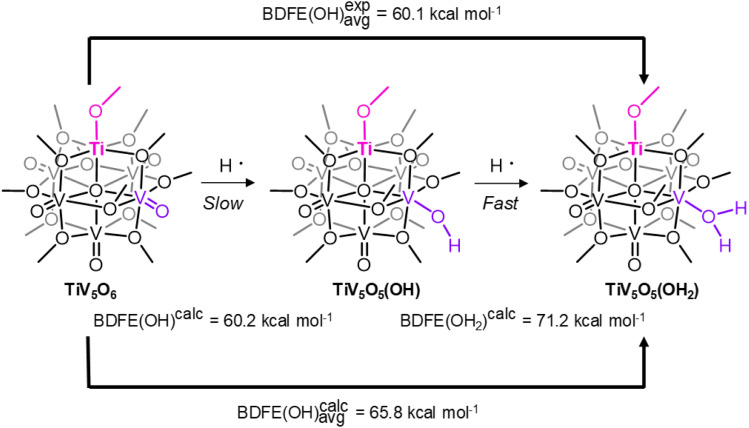
Computational and Experimental BDFE(OH) for Ti-doped POV.

An alternative explanation for the discrepancy in rates of CPET worth consideration is differences in BDFE's for the first and second H-atom of the aquo-ligand. For TiV_5_O_5_(OH_2_) and the reduced product V_6_O_6_(OH_2_)(OCH_3_)_12_^1−^, V_6_O_6_(OH_2_)^1−^, the reported BDFE values for these reduced clusters are averages, due to the instability of the V^IV^-OH proposed intermediate. A strong first BDFE is supported by the lack of comproportionation of oxidized and reduced POV in solution.^23^ If the BDFE of the V–OH is weaker than the BDFE of the V–OH_2_ for either TiV_5_O_5_(OH_2_) or V_6_O_6_(OH_2_)^1−^, then this may result in discrepancies in the reaction rate while maintaining a rate-limiting CPET. Since the V^IV^–OH POV clusters remain elusive, we turn to computational insights into the differences in the first and second O–H bond strengths. The BDFE of the first H-atom (“TiV_5_O_5_(OH)”) and the second H-atom (TiV_5_O_5_(OH_2_)) were calculated for both POV clusters of interest (Scheme 3 and Table 3). The calculated values are larger than the experimentally derived values by 5 kcal mol^−1^. Despite this, there is agreement between the BDFE(O–H)_avg_ values for TiV_5_O_5_(OH_2_) and V_6_O_6_(OH_2_)^1−^ determined experimentally and through computations, and helpful for the observations of the trends for H-atom uptake on the POV.

**Table 3:** Comparison of the theoretical and experimentally determined BDFEs

BDFE (kcal mol^−1^)	TiV_5_O_6_	V_6_O_7_^1−^
V^IV^–OH	60.2	59.1
V^III^–OH_2_	71.2	70.2
Avg.^calc^	65.7	64.7
Avg.^exp^^25,33^	60.1	59.9

Computations predict that the first BDFE is weak, consistent with a rate-limiting H-atom transfer to form the V^IV^–OH moiety for both Ti-doped and homometallic clusters. This is consistent with the mechanism proposed based on experimental findings in our 2H^+^/2e^−^ process to POV clusters.^23^ The second BDFE is found to be much stronger, by ∼10 kcal mol^−1^, which would manifest as a fast step in the reaction that cannot be kinetically resolved through experimentation. In addition, identical averages are computed for TiV_5_O_6_ and V_6_O_7_^1−^. We conclude that while a discrepancy in bond strengths may contribute to the acceleration in reaction rates, our computational model is unable to currently provide appropriate resolution to the BDFE that would support this theory.

Finally, we cannot eliminate the possibility of strong electronic coupling between the H_2_Azo substrate and TiV_5_O_6_. Strong electronic coupling occurs when there is interaction between two molecular orbitals (MOs) resulting in a change of the electron occupancy. Differences in the MOs of TiV_5_O_6_*vs.*V_6_O_7_^1−^ are highlighted in the computational discussion but can also be visualized in the EAS spectrum of the reduced Ti-POV (Fig. 2). For this cluster, we observe the presence of an IVCT band (V(iv) → Ti(iii)). It is possible that the MOs of H_2_Azo are more strongly coupled to the MOs of TiV_5_O_6_ due to the presence of the Ti-ion. The oxidized organic product, Azo, has a weak transition at 440 nm (Fig. S19†) that may couple to TiV_5_O_6_. Evidence of this electronic coupling may be observed by analysis of early timepoints for the reaction of the POV with H_2_Azo *via* EAS (Fig. S20†). When these two substrates are mixed, there is asymmetry observed in the initial *λ*_max_ in comparison to identical reaction conditions for V_6_O_7_^1−^. We note that the strong coupling effects may provide some perspective to the peculiar KIE value, ∼1. Other groups have shown KIE of ≤1 when strong electronic and vibrational coupling occurs, where stronger coupling effects are more profound in deuterated complexes, making the *k*_D_ values similar to *k*_H_ and a KIE of ∼1.^54^

## Conclusions

Understanding the factors that regulate the mechanism of H-atom uptake on metal oxide assemblies afford a unique opportunity to improve the selectivity of products of interfacial reactivity. In this study, we demonstrate the ability to alter the mechanism from stepwise ET–PT to CPET using a Ti^IV^ doped POV cluster by modulating the elementary driving forces of the single proton and electron transfer. By “turning on” the stepwise pathway, the reaction rate is accelerated due to lower activation barriers associated with the PT_limiting_ step. The mechanism of PCET is then changed back to a concerted pathway by switching to a less potent electron reductant. However, despite similar driving forces and activation barriers, an accelerated reaction is observed for the Ti-POV in comparison to the homometallic cluster. The source of the enhanced rates of PCET to TiV_5_O_6_ are discussed, including divergences in BDFE(O–H) of the hydroxide clusters as well as potential strong electronic coupling interactions between the titanium-doped POV and substrate. Ongoing efforts from our laboratory seek to investigate the possibility of strong coupling influences on rate as well as explore the effects of other transition metal ions on the mechanism of PCET in doped POV clusters.

## Experimental

### General considerations

All manipulations were carried out in the absence of water and oxygen using standard Schlenk techniques or in a UniLab MBraun inert atmosphere drybox under a dinitrogen atmosphere. All glassware was oven-dried for a minimum of 2 h and cooled in an evacuated antechamber prior to use in the drybox. Solvents were dried and deoxygenated on a glass contour system (Pure Process Technology, LLC) and stored over 3 Å molecular sieves purchased from Fisher Scientific and activated prior to use. Hydrazobenzene was purchased from TCI America and used as received. 2.5 M *n*-butyllithium in hexanes was purchased from Sigma-Aldrich and used as received. D_2_O was purchased from Cambridge Isotope Laboratories and used as received. POV clusters V_6_O_7_^1−^, TiV_5_O_6_ were prepared according to previously reported procedures.^19,25,45^ 9,10-Dihydrophenazine, 9,10-dihydrophenazine-*d*_2_, and hydrazobenzene-*d*_2_ were generated following literature precedent.^23,55^

^1^H NMR spectra were recorded at 400 MHz or 500 MHz on a Bruker DPX-400 or Bruker DPX-500 spectrometer, respectively, locked on the signal of deuterated solvents. All chemical shifts were reported relative to the peak of the residual H signal in deuterated solvents. CD_3_CN was purchased from Cambridge Isotope Laboratories, degassed by three freeze–pump–thaw cycles, and stored over fully activated 3 Å molecular sieves. All electrochemistry measurements were performed by using a BioLogic SP-150 Potentiostat and acquired with the EC-Lab software (V11.42). Glassy carbon disc (3 mm, CH Instruments, USA) and a platinum wire was used as working and counter electrode, respectively. A nonaqueous Ag/Ag^+^ reference electrode with 1 mM AgNO_3_ and 100 mM [^*n*^Bu_4_N][PF_6_] in acetonitrile (BASi, USA) was used as the reference electrode. All cyclic voltammetry (CV) measurements were carried out at room temperature in a nitrogen-filled glove box and calibrated by Fc^0/+^ couple at 100 mV s^−1^. Electronic absorption measurements were recorded at room temperature in anhydrous acetonitrile in a sealed 1 cm quartz cuvette with an Agilent Cary 60 UV-vis spectrophotometer or an Agilent Cary 3500 spectrophotometer. Kinetic experiments were carried out on an Agilient Cary 60 UV-vis spectrophotometer held at desired temperatures using an Unisoku CoolSpek UV cryostat, as well as an Agilent Cary 3500 UV-vis spectrophotometer held at desired temperatures with an integrated Peltier temperature control system.

### General procedure for second-order reaction kinetics

In a nitrogen-filled glovebox, stock solutions of TiV_5_O_6_ (1.5 mM) and H_2_Phen (15 mM) in de-gassed, dry acetonitrile were prepared in air-tight syringes and removed. All measurements were performed on a KinetAsyst Double-Mixing Stopped-Flow equipped with a cryostat (TgK Scientific). All measurements were taken in single-mixing mode using a photomultiplier tube. Under active N_2_ flow, the syringes were attached to the stopped-flow drive syringes after flushing the system with air-free acetonitrile. Electron transfer from H_2_Phen to TiV_5_O_6_ is instantaneous, resulting in equimolar amounts of H_2_Phen˙^+^ and TiV_5_O_6_^1−^ to be formed in solution, allowing for precise second order reactivity to be observed. The reaction coordinate was monitored at 645 nm to observe the loss of H_2_Phen˙^+^ over time (*λ*_645nm_, *ε* = 1003 M^−1^ cm^−1^, determined *in situ*). Each trial was repeated in triplicate and an averaged trace was obtained for analysis. The procedure was repeated for each new temperature (243–283 K), and the initial concentration of H_2_Phen˙^+^ is in good agreement across the temperature range investigated (Fig. S2†).

Concentration of [H_2_Phen˙^+^] was calculated from the baseline corrected absorbance (*A*) by using the molar absorptivity coefficient (*ε*) at the selected wavelength and the pathlength (*b* = 1 cm) (eqn (2)).

2 [H_2_Phen˙^+^] = *A* × (b × *ε*)^−1^

We note that both cluster reactant (TiV_5_O_6_^1−^) and product (TiV_5_O_5_(OH_2_) absorb at the selected wavelength, but significantly less than the radical (*ε* = 80 M^−1^ cm^−1^ and 100 M^−1^ cm^−1^, respectively). Change in Δ*A* at 0.75 mM cluster concentration <0.02), therefore the overall change in absorbance from the POV cluster is negligible with respect to the radical species (Δ*A*_cluster_ ∼ 0) (Fig. 2).

A plot of [H_2_Phen˙^+^]^−1^*vs.* time reveal a linear relationship for at least 3 half-lives, with excellent fits (*R*^2^ ≥ 0.99). The slope of the line is the second order rate constant (*k*_PT_) for proton transfer from H_2_Phen˙^+^ to TiV_5_O_6_^1−^ at a given temperature. Uncertainties associated with rates were determined by accounting for 10% of the average value.

The rate expression for HAT to TiV_5_O_6_ from H_2_Phen can be described as (eqn (3)):

3 rate = *K*_ET_*k*_PT_[TiV_5_O_6_^1−^][H_2_Phen˙^+^]

where *K*_ET_ is the equilibrium constant for ET from H_2_Phen to TiV_5_O_6_ and *k*_PT_ is the second order rate constant for PT from H_2_Phen˙^+^.

To determine the deuterium kinetic isotope effect (KIE), analogous second order reactions were performed under identical conditions, using the deuterium-labelled reductant species 9,10 dideuterophenazine-*d*_2_ (D_2_Phen). The prepared D_2_Phen used for these reactions was found to be 98% D-labelled by ^1^H NMR spectroscopy. Uncertainties associated with KIE was determined by accounting for 10% of the average value.

### Determination of approximate p*K*_a_ values from the Bordwell equation

To determine approximate p*K*_a_ (p*K*^approx.^_a_) values for TiV_5_O_6_ and H_2_Phen and H_2_Azo, the Borwell equation is employed (eqn (4)):4
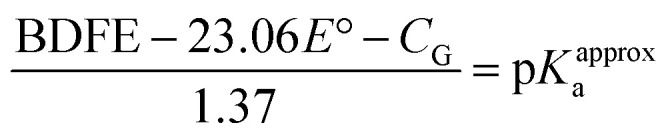
where BDFE is the established bond dissociation free energy of the substrate, *E*° is the reduction potential of the relevant 1 e^−^ process, and *C*_G_ is a constant associated with the solvent. p*K*^approx.^_a_ is 10 and 2 for H_2_Phen and H_2_Azo, respectively.

### General procedure for performing pseudo-first-order reaction kinetics

Pseudo-first-order reaction conditions were used to establish the rate expression for the reaction between the POV clusters (TiV_5_O_6_ and V_6_O_7_^1−^) and the H-atom transfer reagent, H_2_Azo. Using a UV-vis spectrophotometer with temperature controls set to 318 K, reactions between cluster and excess H_2_Azo (100–440 eq.) were tracked by monitoring the absorbance at 1050 nm over the reaction coordinate. Final reductant concentrations were varied from 0.02–0.33 M, with a constant concentration of cluster of 0.75 (TiV_5_O_6_) or 0.60 mM (V_6_O_7_^1−^). Samples of cluster stock solutions in MeCN were loaded in a long-necked quartz cuvette and sealed with a rubber septum and sealed with electrical tape before removing from the glovebox. In a 1 mL syringe (21G needle), a sample of reductant stock solution was measured prior to removal from the glovebox. After equilibrating to 318 K in the spectrophotometer, data acquisition began, and the reductant solution was forcefully injected to ensure efficient sample mixing. Upon the completion of the reaction, the plot of absorbance over time was fit to the following equation by least squares fitting:

5 *A*_*t*_ = *A*_inf_ + (*A*_0_ −*A*_inf_)e^−*k*_obs_*t*^

where *A*_t_ is the absorbance at a given time, *t*, in seconds, *A*_inf_ is the absorbance at the end of the reaction (*t* = infinite), *A*_0_ is the absorbance after reductant injection, and *k*_obs_ is the observed first order rate constant (s^−1^). All reactions to determine *k*_obs_ were performed in triplicate. The good fit found for reaction curves indicated that the rate expression is first order with respect to reductant concentration (Fig. S9, S10 and S12†). Plotting *k*_obs_ as a function of reductant concentration generated a linear plot, meaning that the reaction rate expression is second order overall, such that (eqn (6) and (7)):

6 rate = *k*[TiV_5_O_6_][H_2_Azo]

7 rate = *k*[V_6_O_7_^1−^][H_2_Azo]

The slopes of the resultant *k*_obs_*vs.* [H_2_Azo] plots were normalized for the four (*n* = 4) possible reactive V^V^=O sites on the TiV_5_O_6_ or for the six (*n* = 6) possible reactive V^V^=O sites, as well as the two possible H-atoms which can be transferred from H_2_Azo, in order to determine the second order rate constant, *k*_PCET_ (M^−1^ s ^−1^), such that (eqn (8)):8
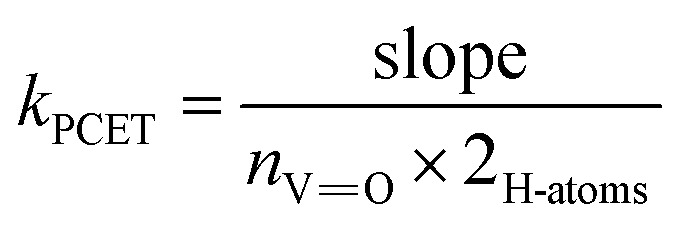


With no observed induction period in the pseudo-first order kinetics traces, the *y*-intercept was held at the origin in all cases. The reported errors are the first significant figure of the difference between the determined slope and the confidence interval maximum. To determine the deuterium kinetic isotope effect (KIE), analogous pseudo-first order reactions were performed under identical conditions, using the deuterium-labelled reductant species Hydrazobenzene-*d*_2_ (D_2_Azo) (Fig. S12–S15†). The prepared D_2_Azo used for these reactions was found to be 99% D-labelled using ^1^H NMR spectroscopy. Uncertainties associated with KIE was determined by accounting for 10% of the average value.

### General considerations for computationally derived free energies

All density functional theory (DFT) calculations were conducted using the Turbomole 7.3 software package. Geometry optimizations were fully converged using the PBE0 hybrid functional, the resolution of the identity approximation (RI-J), the multipole-accelerated RI-J approximation (MA-RIJ), in conjunction with the m4 grid.^56,57^ The Aldrich basis set def2-TZVP was used for all atoms.^58^ Dispersion effects were included using the D3 Grimme correction.^59^ All calculations were performed in gas phase without any constraints. Harmonic frequency calculations were performed to confirm that the optimized structures were stationary points on the potential energy surface. Partition functions were used in the computation of 298 K thermal contributions to free energy employing the usual ideal-gas, rigid-rotor, harmonic oscillator approximation. All frequencies below 100 cm^−1^ were replaced to 100 cm^−1^ when computing Gibbs free energies. Single point calculations including the conductor-like screening model (COSMO) were performed to incorporate solvent effects (tetrahydrofuran, *ε* = 7.58).^60,61^ MultiWFN was used to generate the total and partial density of states, spin density, and molecular orbitals.^62^ Gibbs free reaction energies for elementary PCET steps were calculated according to the following examples (eqn (9)–(11)):POV + H_2_Phen → H-POV + HPhen

9 Δ*G*_PCET_ = *G*_HPOV_ + *G*_HPhen_ − *G*_POV_ − *G*_H_2_Phen_

POV + H_2_Phen → POV^−^ + HPhen^+^

10 Δ*G*_ET_ = *G*_POV_^−^ + *G*_H_2_Phen^+^_ − *G*_POV_ − *G*_H_2_Phen_

POV + H_2_Phen → H-POV^+^ + HPhen^−^

11 Δ*G*_PT_ = *G*_HPOV_^+^ + *G*_HPhen^−^_ − *G*_POV_ − *G*_H_2_Phen_

where POV is either TiV_5_O_6_ or V_6_O_7_^1−^.

## Data availability

All data for this project has been deposited in the main text or ESI.† Additional kinetic traces and computational data can be found in the ESI.†

## Author contributions

S. E. C. executed experiments involving TiV_5_O_6_. S. G. D. performed all computational analysis. M. R. A. W. performed kinetic experiments involving V_6_O_7_^1−^ and H_2_Azo. Stopped flow experiments were operated by N. J. G. P. M. and E. M. M. directed the project. Writing and editing of the manuscript was achieved through contribution of all authors.

## Conflicts of interest

There are no conflicts to declare.

## Supplementary Material

SC-016-D4SC06468B-s001
