# Supplementary material for: Engineering mechanisms of proton-coupled electron transfer to a titanium-substituted polyoxovanadate–alkoxide
Source: Chem Sci. 2025 Jan 7;16(6):2886–97. doi: 10.1039/d4sc06468b (PMC11733765; doi:10.1039/d4sc06468b)
Supplement: SC-016-D4SC06468B-s001 [file SC-016-D4SC06468B-s001.pdf]

Supplementary Information (SI) for Chemical Science.
This journal is © The Royal Society of Chemistry 2025

ELECTRONIC SUPPORTING INFORMATION FILE

Engineering Mechanisms of Proton-Coupled Electron Transfer to a Titanium-substituted Polyoxovanadate-alkoxide

Shannon E. Cooney[a], S. Genevieve Duggan[b,c], M. Rebecca A. Walls[a], Noah J. Gibson[d], James M. Mayer[d], Pere Miro[b,c]*, Ellen M. Matson[a]*

[a]Department of Chemistry, University of Rochester, Rochester, NY 14627, USA

[b]Department of Chemistry, University of Iowa, Iowa City, IA 52240 USA

[c]Department of Chemistry, University of South Dakota, Vermillion, SD 57069 USA

[d]Department of Chemistry, Yale University, New Haven, Connecticut 06520, USA

Contents

Figure S1. Loss of $H_2Phen^{\bullet+}$ as a function of H_2Phen in MeCN at 273 K. 2

Figure S2. Second order plots showing the loss of $H_2Phen^{\bullet+}$ from 243 - 283 K in MeCN. 2

Figure S3. Second order plot showing the loss of $D_2Phen^{\bullet+}$at 298 K. 3

Figure S4. Thermochemical landscape of the reaction of H_2Phen and $\mathbf{TiV_5O_6}$ using M06 functional. ET (vertical), PT (horizontal), and CPET (diagonal). Single point calculations in solvation (THF) at the M06-TZVP//PBE0-TZVP level of theory. Gibbs free energies in kcal mol^{-1}. 3

Figure S5. Thermochemical landscape of the reaction of H_2Phen and $\mathbf{V_6O_7^{1-}}$ using M06 functional. ET (vertical), PT (horizontal), and CPET (diagonal). Single point calculations in solvation (THF) at the M06-TZVP//PBE0-TZVP level of theory. Gibbs free energies in kcal mol^{-1}. 4

Figure S6. Cyclic voltammogram of 1 mM H_2Azo in MeCN, 0.1 M $[^nBu_4N][PF_6]$ electrolyte, 100 mV/s scan rate. 4

Figure S7. ^1H NMR comparing the products of PCET to $\mathbf{TiV_5O_6}$ with H_2Phen (top) and H_2Azo (bottom) in MeCN-d_3 at 298 K. Inset shows Azo peaks are observed at 7.19 (t, 2H), 6.84 (d, 2H), 6.77 (t, 1H) ppm. 5

Figure S8. Scanning kinetic EAS for 0.75 mM $\mathbf{TiV_5O_6}$ + 70 mM H_2Azo in MeCN at 318 K. 5

Figure S9. Low concentration (0.069 – 0.023 M) H_2Azo + 0.75 mM $\mathbf{TiV_5O_6}$ kinetic pseudo-first order traces at 318 K. Concentrations and kobs are listed as insets for each trail. Triplicate trials are reported. Greater deviation of values is due to reaching the lower end of pseudo-first order range of reductant (~100x excess). 6

Figure S10. High concentration (0.107 – 0.322 M) H_2Azo + 0.75 mM $\mathbf{TiV_5O_6}$ kinetic pseudo-first order traces at 318 K. Concentrations and k_{obs} are listed as insets for each trail. Triplicate trials are reported. 6

Figure 11. Duplicate trials at 0.356 M H_2Azo and 2nd order plot with high concentration H_2Azo 7

Figure S12. Pseudo-first order kinetic traces for 0.6 mM $\mathbf{V_6O_7^{1-}}$ + (0.16 - 0.32 M) H_2Azo in MeCN at 318 K. 7

Figure S13. Kinetic traces for the reaction of (0.107 – 0.236 mM) D_2Azo + 0.75 mM $\mathbf{TiV_5O_6}$ at 318 K in MeCN. 8

Figure S14. Plot of k_{obs} vs concentration of D_2Azo from Figure S12. $k_D = 0.55 \pm 0.03$ M^{-1} s^{-1} at 318 K. 8

Figure S15. Kinetic traces for the reaction of (0.16 – 0.28 mM) D_2Azo + 0.6 mM $\mathbf{V_6O_7^{1-}}$ in MeCN at 318 K. 9

Figure S16. Plot of the observed rate constant (k_{obs}) and concentration of D_2Azo from Figure S15, 318 K in MeCN. $k_D = (6.0 \pm 0.5) \times 10^{-4}$ M^{-1} s^{-1}. 10

Figure S17. Multi temperature (308 - 348 K) kinetic traces for the reaction of 0.24 M H_2Azo + 0.75 mM $\mathbf{TiV_5O_6}$ in MeCN. Triplicate trials presented. 11

Figure S18. Multi temperature (308 - 348 K) kinetic traces for the reaction of 0.24 M H_2Azo + 0.6 mM $\mathbf{V_6O_7^{1-}}$ in MeCN. Triplicate trials presented 12

Figure S19. EAS of H_2Azo and Azo in MeCN at 298 K in MeCN, normalized to concentration. 12

Figure S20. (Left) EAS of 0.75 mM $\mathbf{TiV_5O_6}$ + 70 mM H_2Azo in MeCN at 298 K, initial scan after injection of reductant shows peak asymmetry at 410 nm. (Right) EAS of 0.6 mM $\mathbf{V_6O_7^{1-}}$ + 0.06 M H_2Azo in MeCN at 318 K, no peak asymmetry is observed. 13

The online repository link (iochembd) for all of our .xyz 13

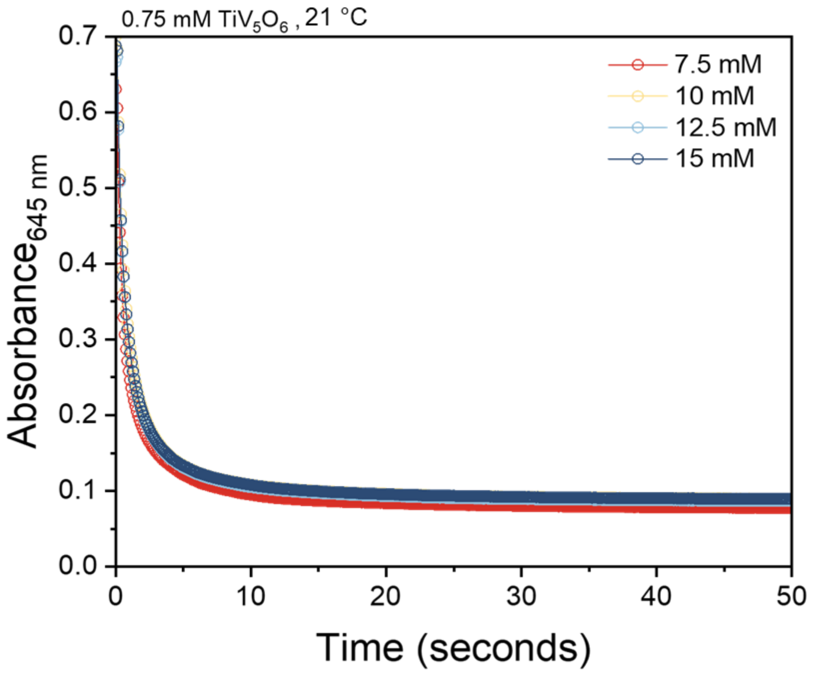

Figure S1. Loss of $H_2Phen^{\bullet+}$ as a function of H_2Phen in MeCN at 273 K.

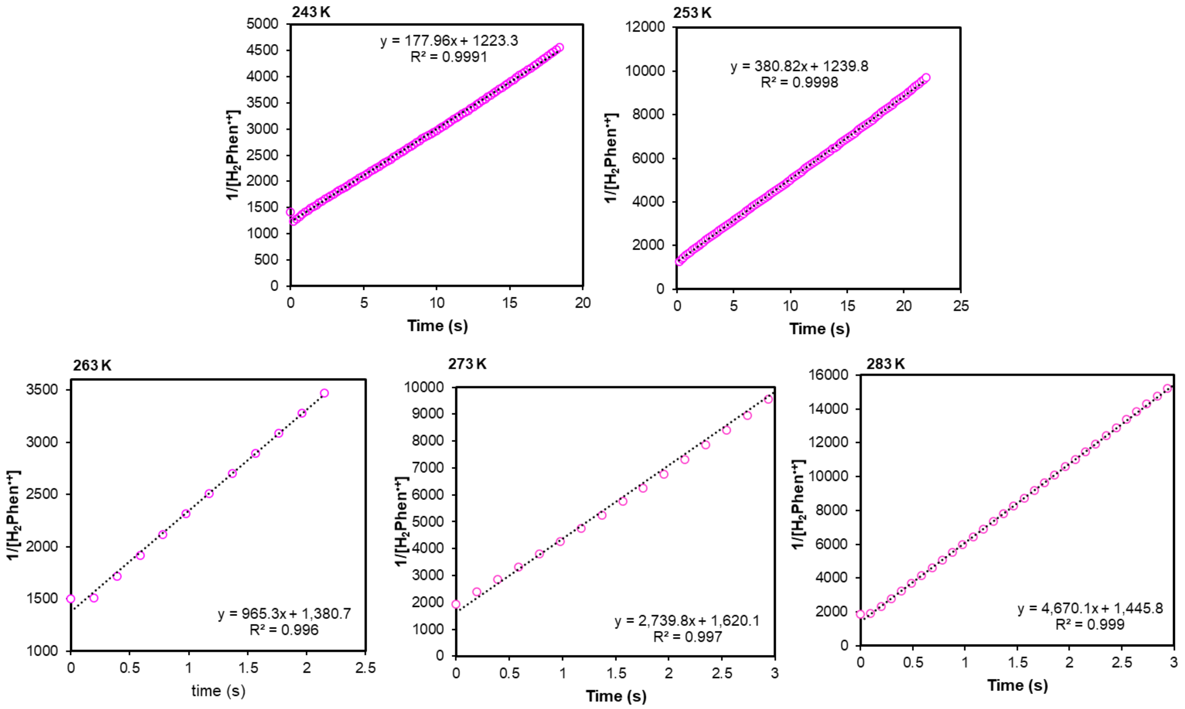

Average intercept from trials 243 – 283 K is 1312.9 M^{-1} $[H_2Phen^{\bullet+}]_{initial}^{average}$ = 0.00076 M H_2Phen^{+}

Figure S2. Second order plots showing the loss of $H_2Phen^{\bullet+}$ from 243 - 283 K in MeCN.

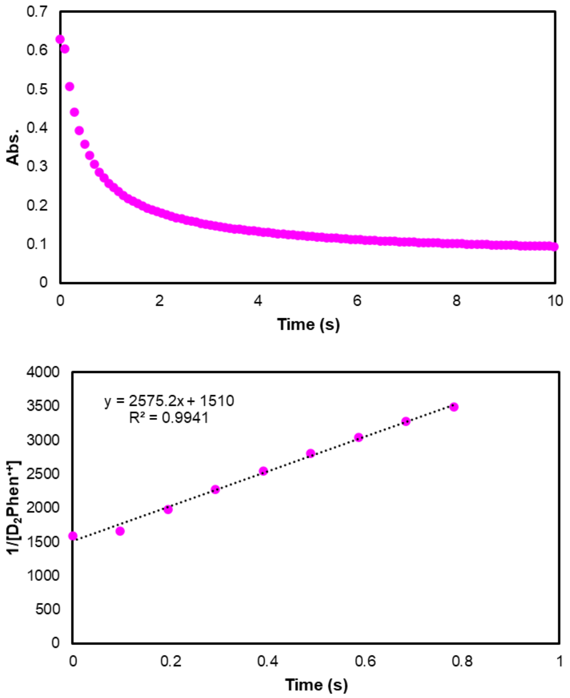

Figure S3. Second order plot showing the loss of $D_2Phen^{\bullet+}$at 298 K.

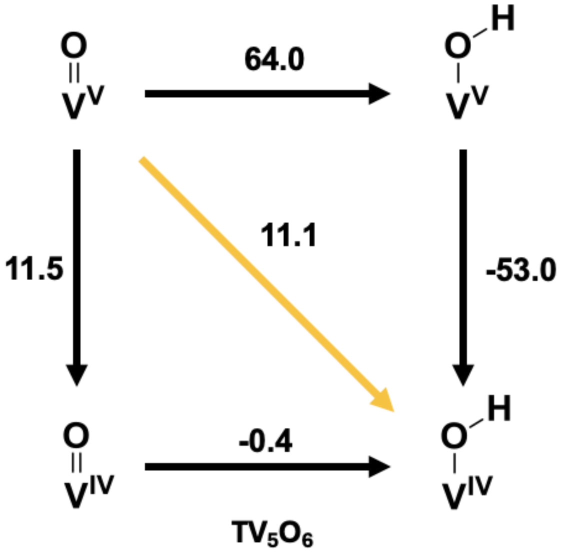

Figure S4. Thermochemical landscape of the reaction of H_2Phen and **TiV_5O_6** using M06 functional. ET (vertical), PT (horizontal), and CPET (diagonal). Single point calculations in solvation (THF) at the M06-TZVP//PBE0-TZVP level of theory. Gibbs free energies in kcal mol^{-1}.

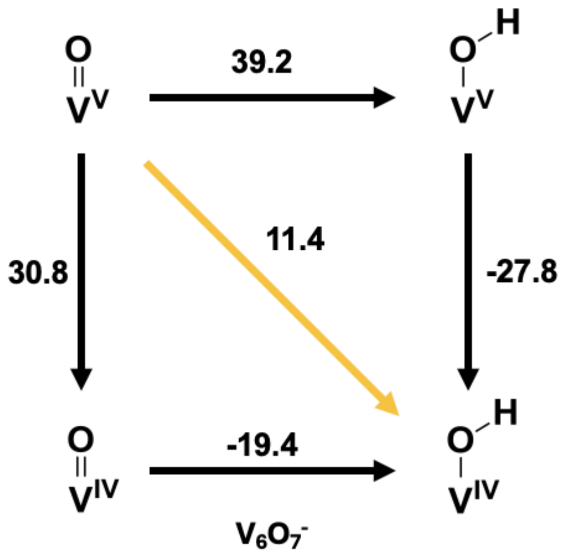

Figure S5. Thermochemical landscape of the reaction of H_2Phen and $\mathbf{V_6O_7}^{1-}$ using M06 functional. ET (vertical), PT (horizontal), and CPET (diagonal). Single point calculations in solvation (THF) at the M06-TZVP//PBE0-TZVP level of theory. Gibbs free energies in kcal mol^{-1}.

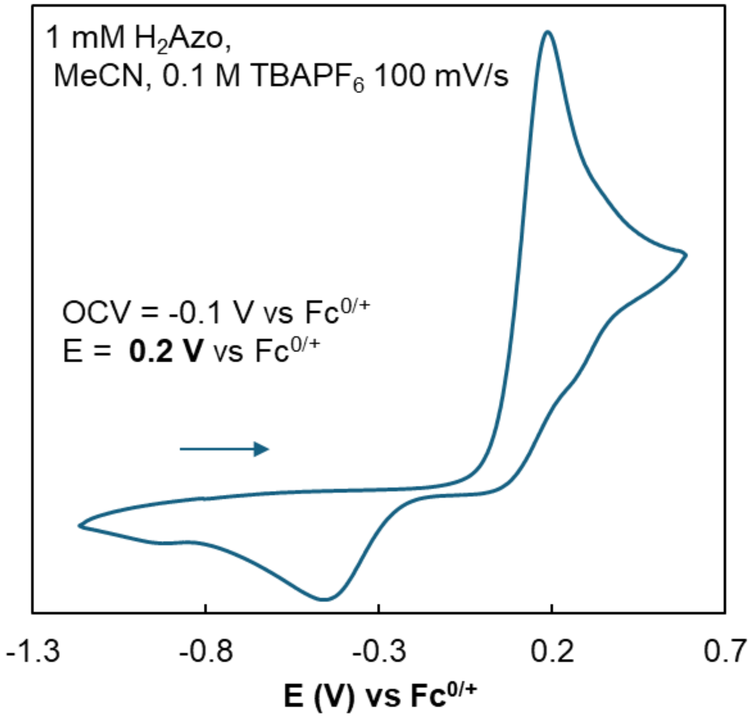

Figure S6. Cyclic voltammogram of 1 mM H_2Azo in MeCN, 0.1 M $[^nBu_4N][PF_6]$ electrolyte, 100 mV/s scan rate.

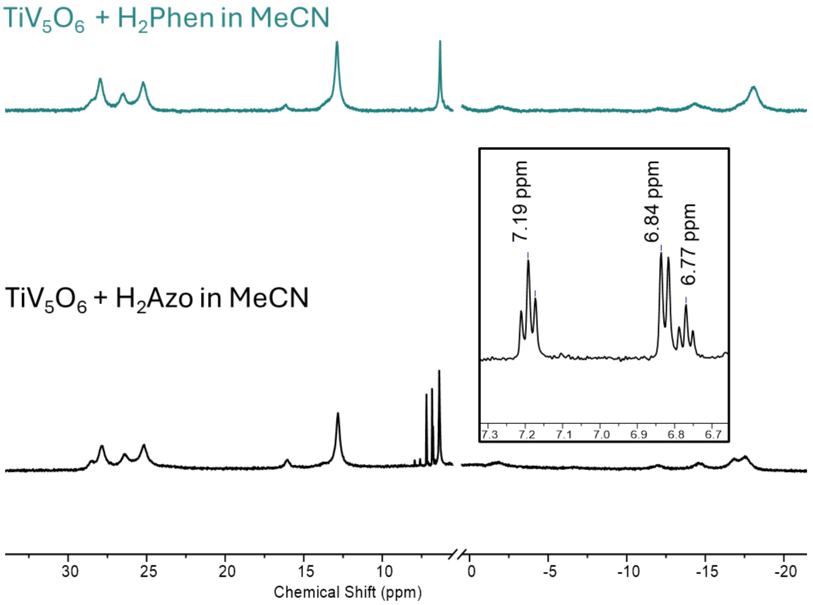

Figure S7. ^{1}H NMR comparing the products of PCET to **TiV_5O_6** with H_2Phen (top) and H_2Azo (bottom) in MeCN-d_3 at 298 K. Inset shows Azo peaks are observed at 7.19 (t, 2H), 6.84 (d, 2H), 6.77 (t, 1H) ppm.

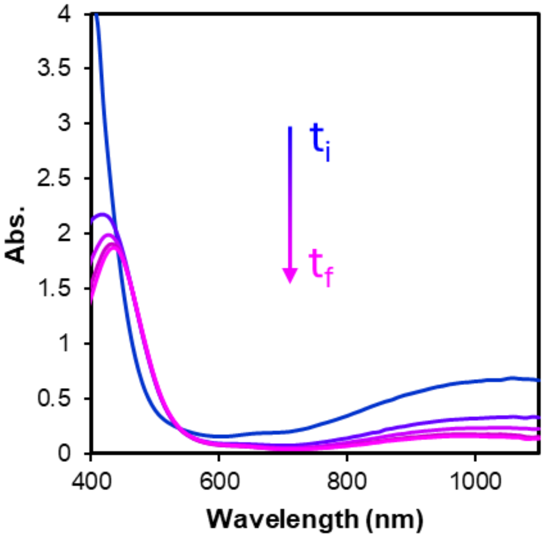

Figure S8. Scanning kinetic EAS for 0.75 mM **TiV_5O_6** + 70 mM H_2Azo in MeCN at 318 K.

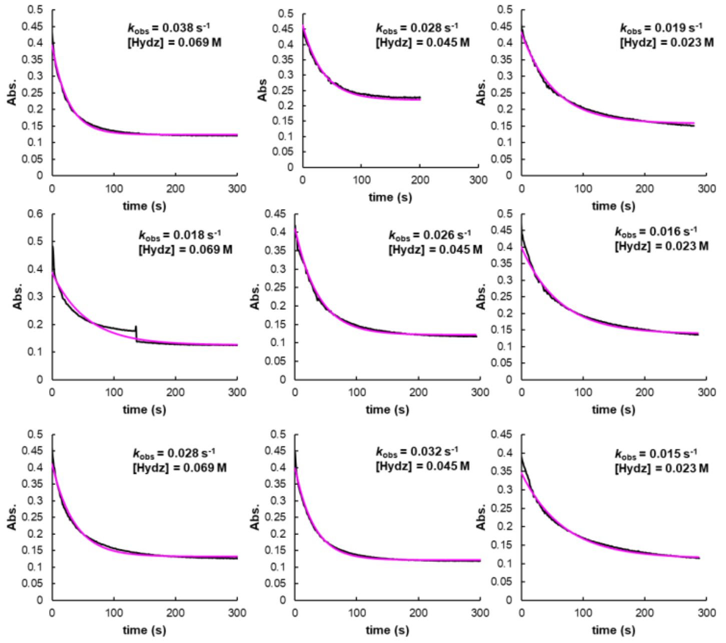

Figure S9. Low concentration (0.069 – 0.023 M) H_2Azo + 0.75 mM **TiV_5O_6** kinetic pseudo-first order traces at 318 K. Concentrations and kobs are listed as insets for each trail. Triplicate trials are reported. Greater deviation of values is due to reaching the lower end of pseudo-first order range of reductant (~100x excess).

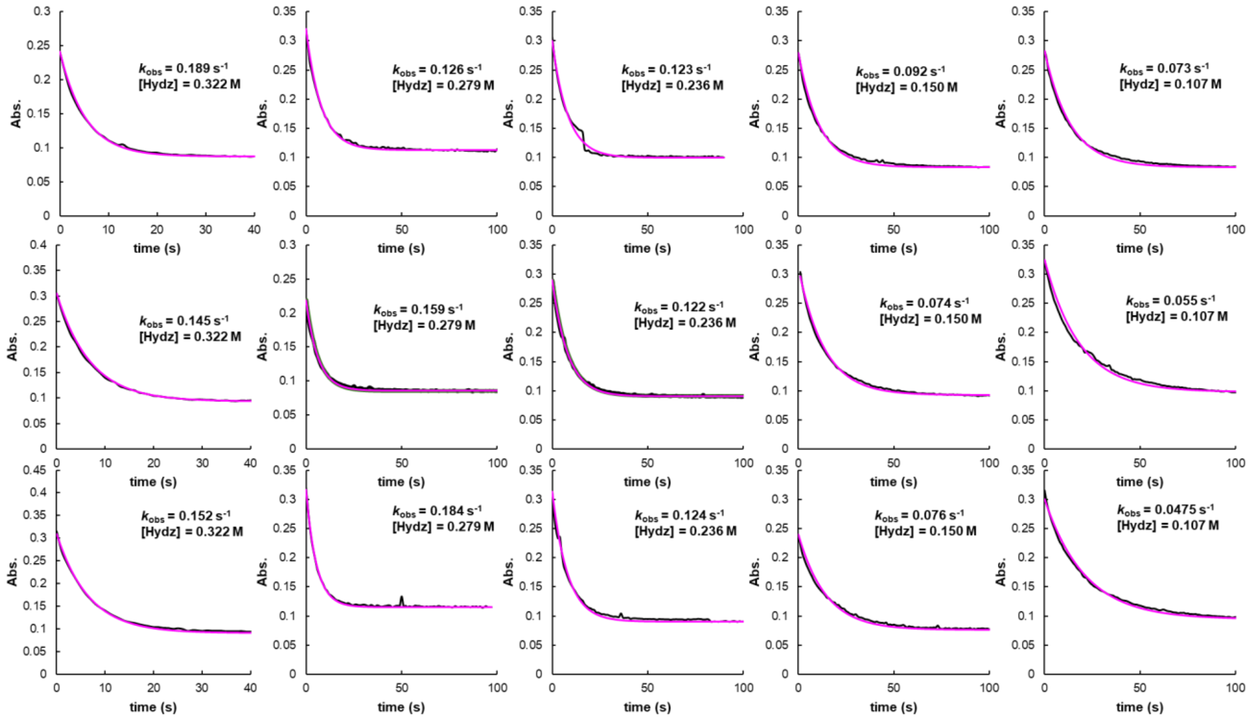

Figure S10. High concentration (0.107 – 0.322 M) H_2Azo + 0.75 mM **TiV_5O_6** kinetic pseudo-first order traces at 318 K. Concentrations and k_{obs} are listed as insets for each trail. Triplicate trials are reported.

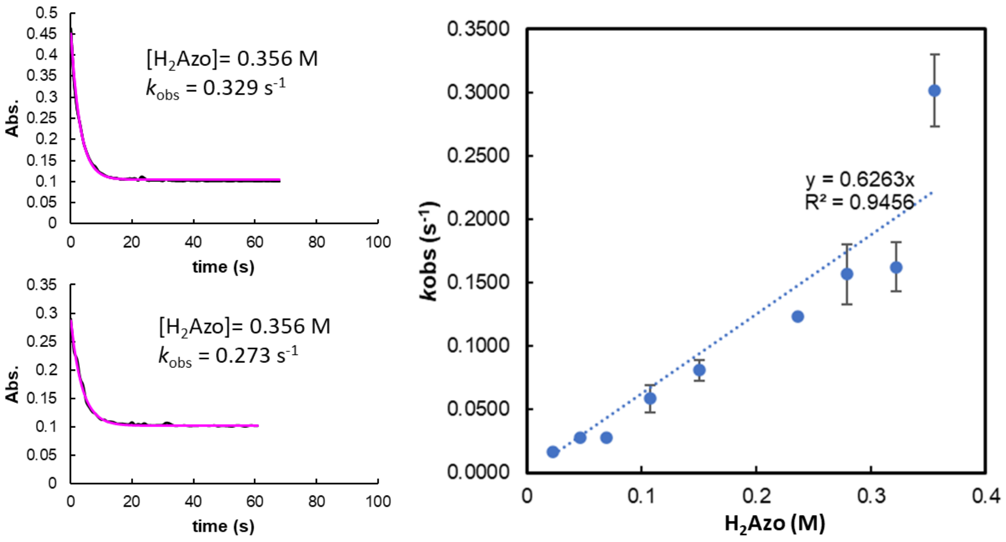

Figure 11. Duplicate trials at 0.356 M H_2Azo and 2nd order plot with high concentration. No leveling effect is observed at high concentration of H_2Azo.

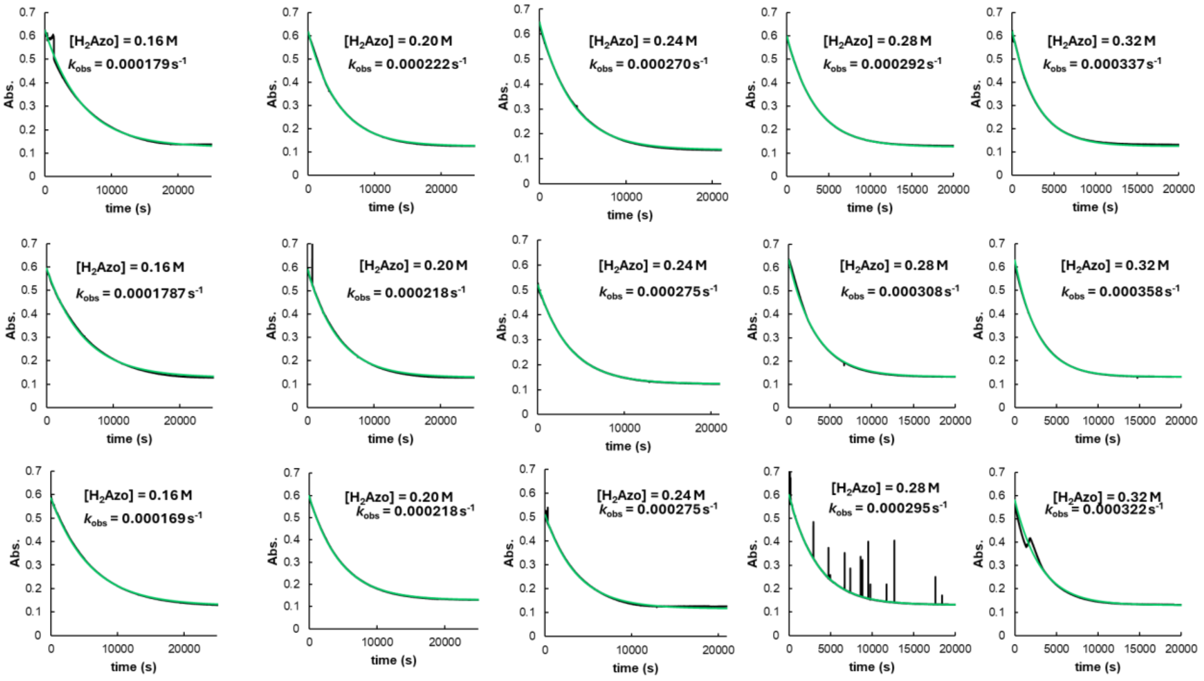

Figure S12. Pseudo-first order kinetic traces for 0.6 mM $\mathbf{V_6O_7^{1-}}$ + (0.16 - 0.32 M) H_2Azo in MeCN at 318 K.

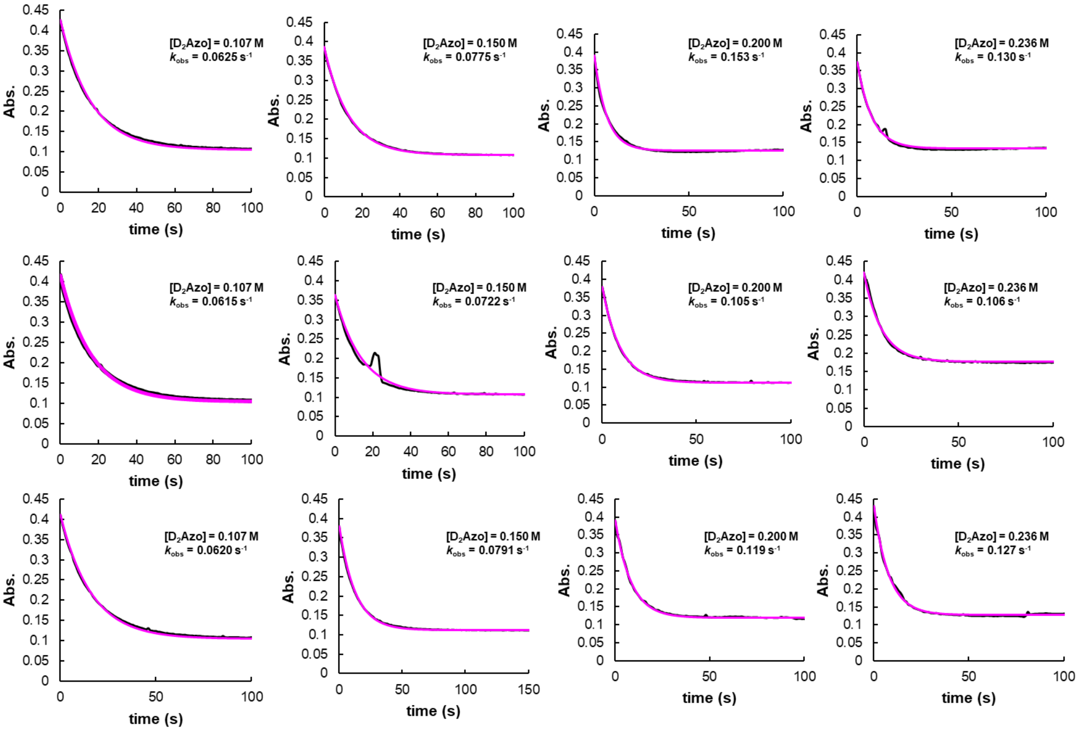

Figure S13. Kinetic traces for the reaction of (0.107 – 0.236 mM) D_2Azo + 0.75 mM **TiV_5O_6** at 318 K in MeCN.

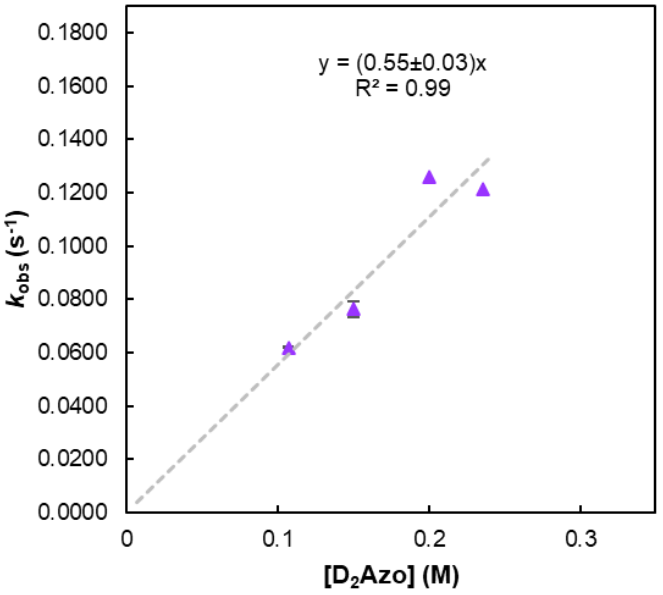

Figure S14. Plot of k_{obs} vs concentration of D_2Azo from Figure S12. $k_D = 0.55 \pm 0.03$ M^{-1} s^{-1} at 318 K.

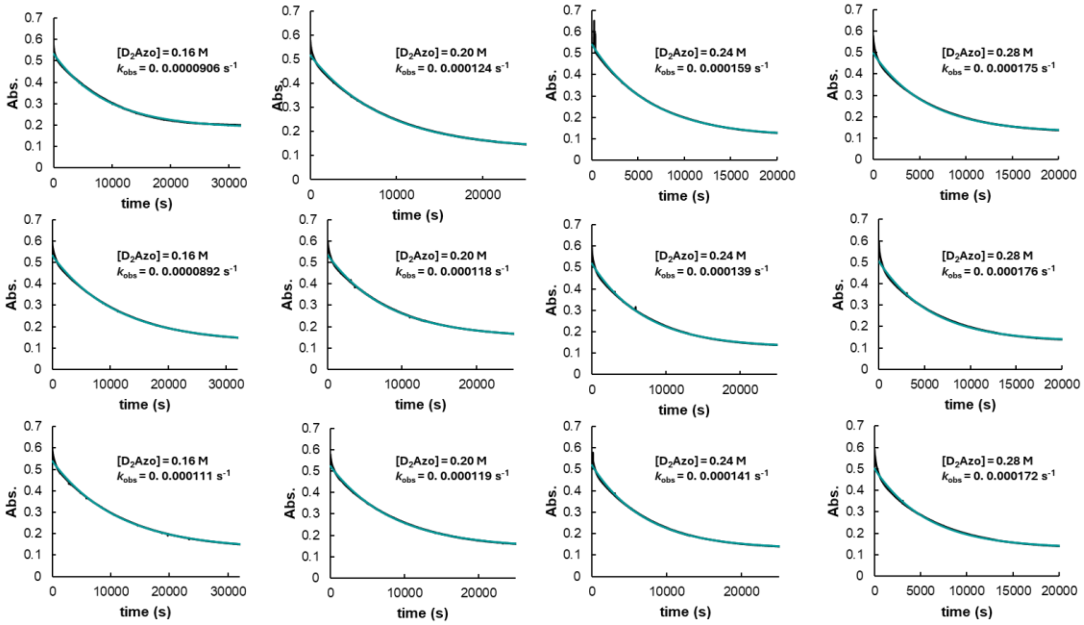

Figure S15. Kinetic traces for the reaction of (0.16 – 0.28 mM) D_2Azo + 0.6 mM $\mathbf{V_6O_7}^{\mathbf{1-}}$ in MeCN at 318 K.

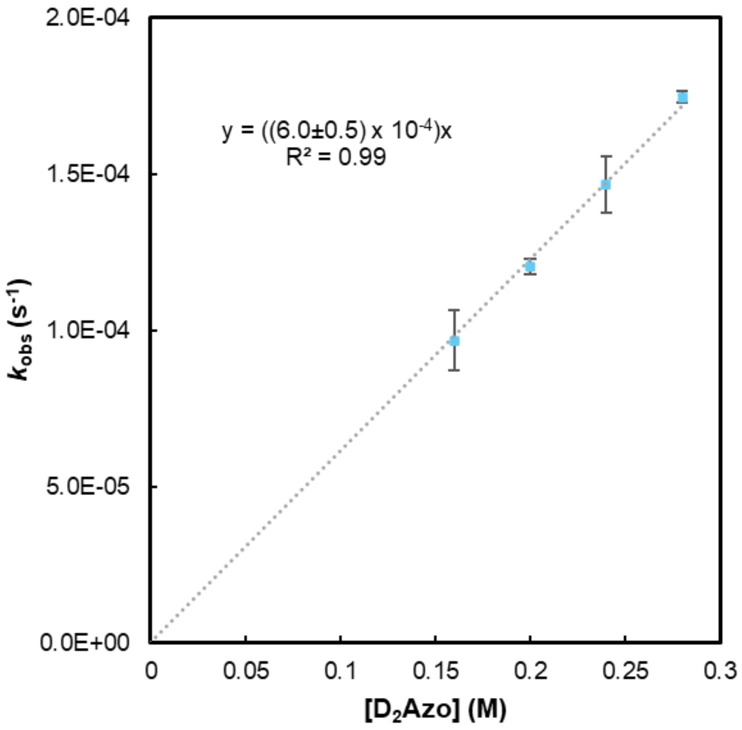

Figure S16. Plot of the observed rate constant (k_{obs}) and concentration of D_2Azo from Figure S15, 318 K in MeCN. k_D = (6.0 ± 0.5) x 10^{-4} M^{-1} s^{-1}.

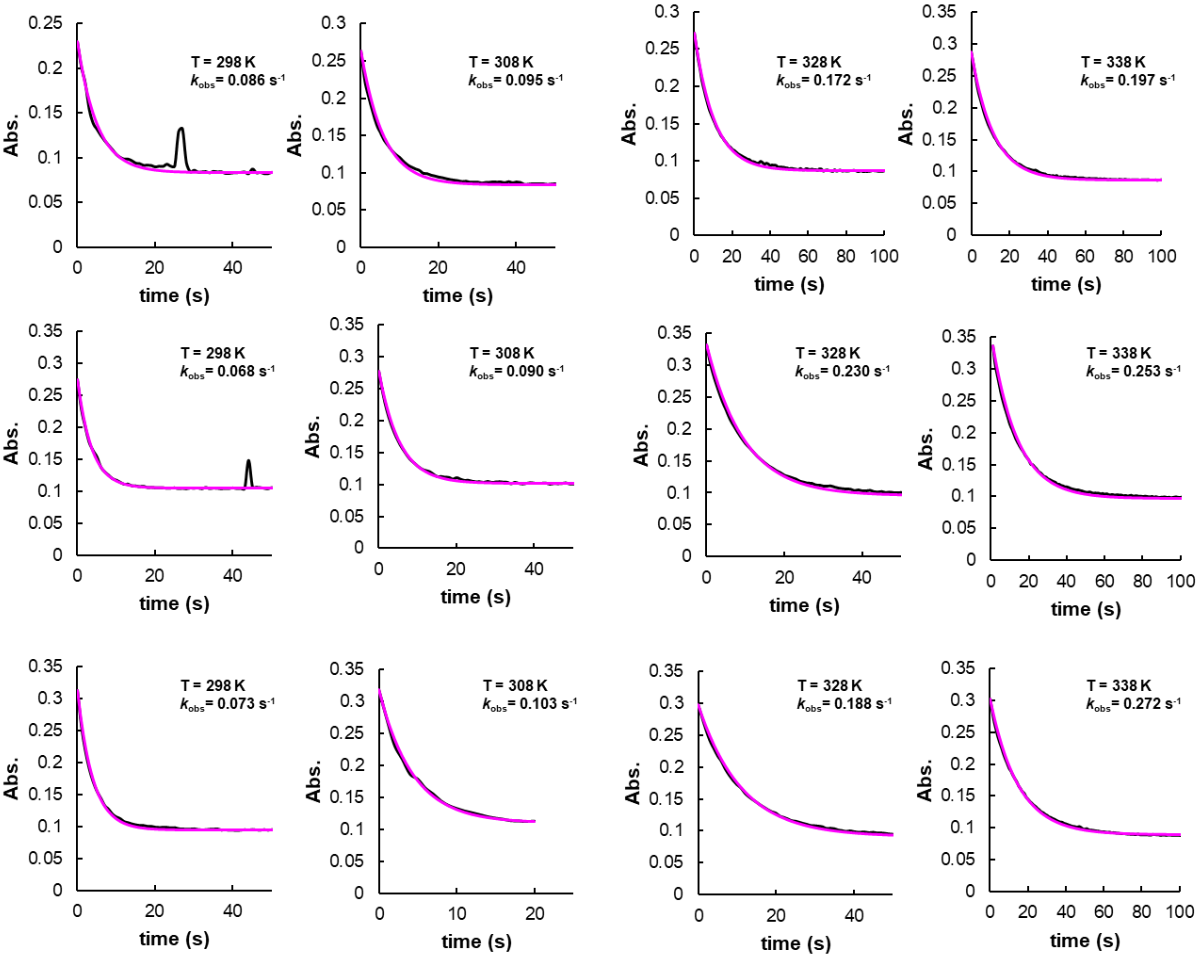

Figure S17. Multi temperature (308 - 348 K) kinetic traces for the reaction of 0.24 M H_2Azo + 0.75 mM **TiV_5O_6** in MeCN. Triplicate trials presented.

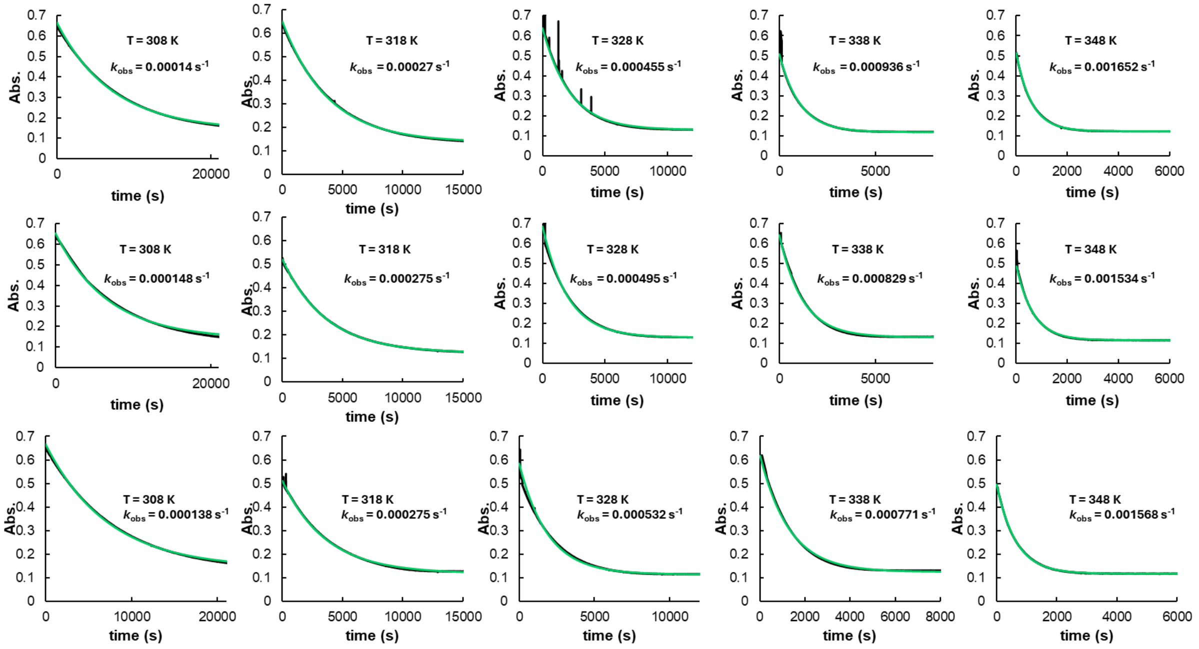

Figure S18. Multi temperature (308 - 348 K) kinetic traces for the reaction of 0.24 M H_2Azo + 0.6 mM $\mathbf{V_6O_7}^{1-}$ in MeCN. Triplicate trials presented

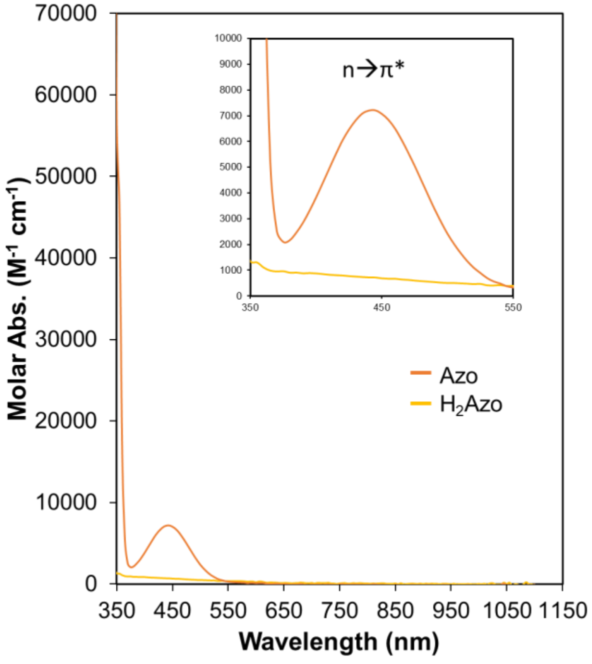

Figure S19. EAS of H_2Azo and Azo in MeCN at 298 K in MeCN, normalized to concentration.

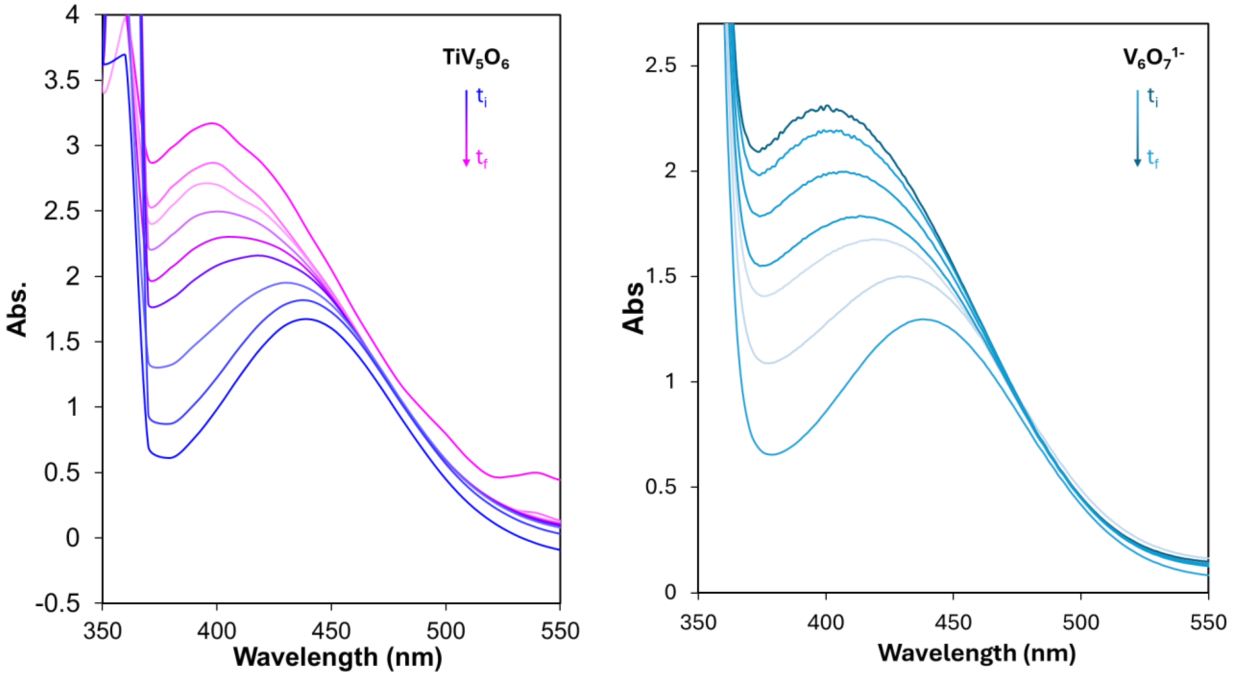

Figure S20. (Left) EAS of 0.75 mM $\mathbf{TiV_5O_6}$ + 70 mM H_2Azo in MeCN at 298 K, initial scan after injection of reductant shows peak asymmetry at 410 nm. (Right) EAS of 0.6 mM $\mathbf{V_6O_7^{1-}}$ + 0.06 M H_2Azo in MeCN at 318 K, no peak asymmetry is observed.

The online repository link (iochembd) for all of our .xyz files are available at:

https://iochem-bd.bsc.es/browse/review-collection/100/328362/2a138d1354110690f12c8a59
